# Fosmid library end sequencing reveals a rarely known genome structure of marine shrimp *Penaeus monodon*

**DOI:** 10.1186/1471-2164-12-242

**Published:** 2011-05-17

**Authors:** Shiao-Wei Huang, You-Yu Lin, En-Min You, Tze-Tze Liu, Hung-Yu Shu, Keh-Ming Wu, Shih-Feng Tsai, Chu-Fang Lo, Guang-Hsiung Kou, Gwo-Chin Ma, Ming Chen, Dongying Wu, Takashi Aoki, Ikuo Hirono, Hon-Tsen Yu

**Affiliations:** 1 Institute of Zoology and Department of Life Science, National Taiwan University, Taipei 10617, Taiwan, ROC; 2 Genome Research Center, National Yang-Ming University, Taipei 112, Taiwan, ROC; 3 Division of Molecular and Genomic Medicine, National Health Research Institutes, Miaoli County 350, Taiwan, ROC; 4 Center for Medical Genetics, and Genetics Laboratory, Department of Medical Research, Changhua Christian Hospital, Changhua 500, Taiwan, ROC; 5 Department of Obstertrics and Gynecology, College of Medicine, National Taiwan University, Taipei 106, Taiwan; 6 Department of Energy Joint Genome Institute, Walnut Creek, California 94598, USA; 7 Department of Evolution and Ecology, University of California Davis Genome Center, University of California, Davis, California 95616, USA; 8 Laboratory of Genome Science, Tokyo University of Marine Science and Technology, Konan 4-5-7 Minato-ku, Tokyo 108-8477, Japan

## Abstract

**Background:**

The black tiger shrimp (*Penaeus monodon*) is one of the most important aquaculture species in the world, representing the crustacean lineage which possesses the greatest species diversity among marine invertebrates. Yet, we barely know anything about their genomic structure. To understand the organization and evolution of the *P. monodon *genome, a fosmid library consisting of 288,000 colonies and was constructed, equivalent to 5.3-fold coverage of the 2.17 Gb genome. Approximately 11.1 Mb of fosmid end sequences (FESs) from 20,926 non-redundant reads representing 0.45% of the *P. monodon *genome were obtained for repetitive and protein-coding sequence analyses.

**Results:**

We found that microsatellite sequences were highly abundant in the *P. monodon *genome, comprising 8.3% of the total length. The density and the average length of microsatellites were evidently higher in comparison to those of other taxa. AT-rich microsatellite motifs, especially poly (AT) and poly (AAT), were the most abundant. High abundance of microsatellite sequences were also found in the transcribed regions. Furthermore, *via *self-BlastN analysis we identified 103 novel repetitive element families which were categorized into four groups, *i.e*., 33 WSSV-like repeats, 14 retrotransposons, 5 gene-like repeats, and 51 unannotated repeats. Overall, various types of repeats comprise 51.18% of the *P. monodon *genome in length. Approximately 7.4% of the FESs contained protein-coding sequences, and the Inhibitor of Apoptosis Protein (IAP) gene and the Innexin 3 gene homologues appear to be present in high abundance in the *P. monodon *genome.

**Conclusions:**

The redundancy of various repeat types in the *P. monodon *genome illustrates its highly repetitive nature. In particular, long and dense microsatellite sequences as well as abundant WSSV-like sequences highlight the uniqueness of genome organization of penaeid shrimp from those of other taxa. These results provide substantial improvement to our current knowledge not only for shrimp but also for marine crustaceans of large genome size.

## Background

Crustaceans (lobster, shrimp, crab, *etc*.), a remarkable group of organisms filling up all types of habitats in the ocean with a wide array of adaptations, possess the greatest species diversity among marine animals. They are not only abundant in number, but also are among the most commercially exploited food species for human consumption [1]. Given their primarily aquatic habitats, however, they are not as well studied as insects, their terrestrial arthropod relatives.

The tiger shrimp (*Penaeus monodon*) has been one of the most important captured and cultured marine crustaceans in the world, especially in the Indo-Pacific region [1,2]. However, the tiger shrimp industry has been plagued by viral diseases [3-5], resulting in substantial economic losses. Developments in shrimp genomics have been limited although a reasonably good EST database is available (Penaeus Genome Database; http://sysbio.iis.sinica.edu.tw/page/) [6]. A genomic analysis for the tiger shrimp will make a key contribution to deciphering the evolutionary history representing the crustacean lineages, especially those living in the ocean. The information contained in the genomic sequences will also benefit the shrimp industry by offering genomic tools to fend off the viral diseases and to improve the breeding program.

The genome size of the penaeid shrimp is estimated to be 2/3 of the human genome [7] and thus an order of magnitude lager than the model invertebrates, *Caenorhabditis elegans *and *Drosophila melanogaster*. Concerning their larger genome size than other invertebrates, we are most interested in knowing what the makeup of genomic DNA in the tiger shrimp genome is. Our initial attempt to sequence a few fosmid clones was hindered by an unusual high percentage of failure in sequencing reactions and by difficulties in assembling contigs, rousing suspicion that the shrimp genome is extraordinarily repetitive in nature. Consequently we set out to have a glimpse of the genomic structure by sequencing ends of fosmid clones. The results would offer insights to whole genome sequencing with appropriate and effective strategies. To achieve this aim, we constructed a *P. monodon *fosmid library from a female shrimp and made an initial analysis of 20,926 high-quality end sequences, a total of 11,114,786 bp representing 0.45% of the whole genome. The results provide substantial improvement to our current knowledge not only for shrimp but also for the genomic structure of invertebrates with large genomes.

## Results

### Estimation of the *P. monodon *genome size

The genome size of *P. monodon *has never been determined experimentally and therefore we measured DNA content of hemocytes of *P. monodon *with flow cytometry, using human lymphocytes as standardized control. In addition, the white shrimp (*P. vannamei*) genome, whose size is known, was used as a reference. The 1*C *nuclear DNA content of *P. monodon *was estimated to be ~72.2% of the human genome, *i.e*., ~ 2.53 pg DNA per nucleus or 2.17×10^9^bp per haploid genome. We also obtained the DNA content of *P. vannamei *to be 71.5% of the human genome (Additional file 1), which is consistent with the value previously reported by Chow *et al*. [7]. The 1*C *value of *P. monodon *is close to those previously reported four other penaeid shrimp species (2.37-2.51 pg for *P. aztecus*, *P. duorarum*, *P. vannamei*, and *P. setiferus*; see Chow *et al*. [7]).

### Construction and characterization of the fosmid library

The constructed *P. monodon *fosmid library consists of a total of 288,000 clones arrayed in 750×384-well microtiter plates. To evaluate the average insert size, 111 clones were randomly selected from the fosmid library and analyzed with *Not*I. The average insert sizes (40.8 ± 3.6 kb) were close to the expected 40 kb (Additional file 2). Therefore, the *P. monodon *fosmid library covers 5.3× haploid genome equivalents based on an estimate of 2.17×10^9^bp per haploid genome.

### Fosmid-end-sequence (FES) analysis

A total of 20,926 high-quality FESs (GenBank accession number JJ726384-JJ747309) with read lengths of ≥100 bp (Additional file 3) were obtained from 11,850 fosmid clones. Of the 11,850 fosmid clones, 9,072 clones had both end sequences present in our FESs. The length of the FESs ranged from 100 bp to 861 bp, with an average read length of 531 bp. A total of 11,114,786 bp of genomic sequences were generated from this study, representing approximately 0.45% of the *P. monodon *genome. The *P. monodon *genome appeared to be AT-rich, with GC content of 45.88%. This is the first estimate of GC content in a marine shrimp.

### Repetitive sequence analysis

Repetitive sequences comprise an important part of eukaryotic genomes, and each species has its own characteristic repetitive sequences. The overall constitution of repetitive elements in the *P. monodon *genome was assessed by RepeatMasker. Of the 20,926 fosmid end reads, 49.82% (10,425/20,926) contained repeats (against *A. gambiae *repeat database). In terms of lengths, 15.49% and 15.44% of base pairs were repeatmasked against *D. melanogaster *and *A. gambiae *repeat database, respectively (Table 1).

**Table 1:** Characterization of repeat types by RepeatMasker*

	*D. melanogaster*			*A. gambiae*		
		
Type	# Hits	Length (bp)	% Bases	# Hits	Length (bp)	% Bases
**(1) Interspersed repeats**						
**(i) *Retrotransposon***	380	45,036	0.405%	870	94,532	0.851%
Non-LTR elements	154	14,584	0.131%	151	16,570	0.149%
L2/CR1/Rex	1	67	0.001%	28	1,817	0.016%
R1/LOA/Jockey	55	4,375	0.039%	122	14,723	0.132%
RTE/Bov-B	-	-	-	1	30	0.000%
LTR elements	226	30,452	0.274%	719	77,962	0.701%
Ty1/Copia	3	238	0.002%	-	-	-
Gypsy/DIRS1	181	27,142	0.244%	718	77,743	0.699%
**(ii) *DNA transposons***	53	4,302	0.039%	154	14,817	0.133%
hobo-Activator	3	363	0.003%	-	-	-
Others	40	3,364	0.030%	40	2,324	0.021%
**(iii) *Unclassified***	48	4,816	0.043%	5	309	0.003%

**Total interspersed repeats**	**481**	**54,154**	**0.487%**	**1,029**	**109,658**	**0.987%**

**(2) Small RNAs**	1,351	422,537	3.802%	1,274	414,435	3.729%
**(3) Simple repeats**	10,282	858,898	7.728%	9,756	807,927	7.269%
**(4) Low complexity repeats**	4,258	386,684	3.479%	4,257	387,159	3.483%
**Total repetitive sequences**		**1,721,824**	**15.491%**		**1,718,177**	**15.458%**

*The repeat databases of *D. melanogaster and A. gambiae *were used.

In spite of similar proportions of repetitive sequences masked by two different, *i.e*., *D. melanogaster *and *A. gambiae*, databases, the lengths allocated in major repeat types were different (Table 1). The length of transposable elements (both retrotransposons and DNA transposons) masked in the *D. melanogaster *database (54,154 bp) was much less than in the *A. gambiae *database (109,658 bp), while the length of simple repeats masked in the *D. melanogaster *database (858,898 bp) was larger than in the *A. gambiae *database (807,927 bp).

Of all repeat types, simple repeats were the most abundant type, identified in approximately 7.5% (7.73% against *D. melanogaster *and 7.27% against *A. gambiae*) of the total 11,114,786-bp FESs and accounting for nearly half of the repetitive sequences. Low complexity repeats (3.48% average over two databases) and small RNAs (3.77% average over 2 databases) were two other abundant repeat types (Table 1). Interspersed repeats (mainly retrotransposons and DNA transposons) were the least abundant (0.74% average over two databases), accounting for only a small fraction (4.76%) of repetitive sequences in length. Among transposable elements, long terminal repeat (LTR) retrotransposons were the most abundant, followed by non-LTR retrotransposons and DNA transposons. Among the LTR retrotransposons, the *gypsy*-type ranked first, which was the only LTR element identified by RepeatMasker using the *A. gambiae *repeat database.

### Frequency and relative abundance of microsatellites in *P. monodon*

In analyzing the components of repetitive sequences, we noticed that simple repeats, in which microsatellites are included, comprise a significant proportion (~7.5%) of tiger shrimp genome and account for most of the repeat types. To further characterize the distribution and constitution of microsatellites in the *P. monodon *genome, 11,114,786-bp fosmid ends were analyzed by Tandem Repeat Finder. Nearly one-third (32.4%) of the end sequence reads contained microsatellites, and a total of 8,441 microsatellite loci comprising 8.3% of 11,114,786-bp fosmid ends were identified. The microsatellite loci are AT biased, with an A/T content of 61.7%.

**Figure 1:**
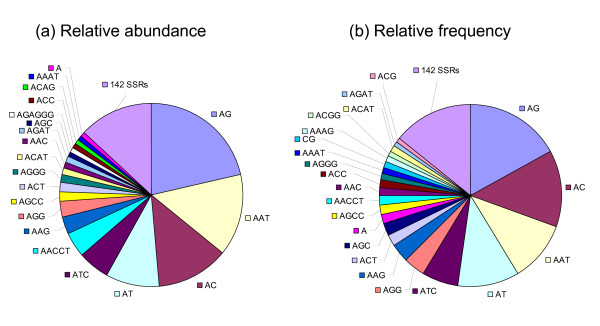
**Relative abundance by base-pair (a) and relative frequencies by loci (b) of top 20 microsatellite classes present in total fosmid end sequences**. 142 SSRs: remaining 142 simple sequence repeat motifs.

Of all microsatellite classes, di- (44.3%) and tri-nucleotide repeats (31.0%) comprise more than 70% of their total length. In decreasing order, the 20 most frequently occurring microsatellites are AG, AC, AAT, AT, ATC, AGG, AAG, ACT, AGC, A, AGCC, AACCT, AAC, ACC, AGGG, AAAT, CG, AAAG, ACGG, and ACAT (Table 2 Figure 1), including all 4 dinucleotide motifs and almost all 10 trinucleotide motifs except ACG and CCG, constituent of 85.8% of microsatellite motifs identified.

**Table 2:** Characterization of microsatellites in the *P. monodon *genome^a^

Motif	Unit	Counts*^b^*	# FES*^c^*	% FES*^d^*	Bases (bp)*^e^*	Bases (%)*^f^*	Max length*^g^*	Mean length*^h^*	STD length*^i^*	Mean repeat no.*^j^*	RA (%)*^k^*	RF (%)*^l^*
A	1	159	158	0.76	7,467	0.07	376	46.96	35.63	46.96	0.81	1.88
AC	2	1153	1095	5.23	118,430	1.07	783	102.71	93.67	51.36	12.85	13.66
AG	2	1424	1395	6.67	198,119	1.78	763	139.13	104.88	69.56	21.50	16.87
AT	2	912	896	4.28	87,832	0.79	604	96.31	70.28	48.15	9.53	10.80
CG	2	91	91	0.43	3,995	0.04	142	43.90	10.94	21.95	0.43	1.08
AAC	3	125	114	0.54	10,390	0.09	457	83.12	65.03	27.71	1.13	1.48
AAG	3	265	255	1.22	27,823	0.25	720	104.99	111.32	35.00	3.02	3.14
AAT	3	915	881	4.21	130,973	1.18	751	143.14	121.98	47.71	14.21	10.84
ACC	3	111	108	0.52	7,814	0.07	584	70.40	77.28	23.47	0.85	1.32
ACG	3	69	67	0.32	5,163	0.05	184	74.83	32.98	24.94	0.56	0.82
ACT	3	182	181	0.86	13,809	0.12	659	75.87	72.91	25.29	1.50	2.16
AGC	3	169	163	0.78	8,098	0.07	159	47.92	19.98	15.97	0.88	2.00
AGG	3	333	321	1.53	25,140	0.23	422	75.50	68.45	25.17	2.73	3.95
ATC	3	545	536	2.56	50,116	0.45	596	91.96	66.43	30.65	5.44	6.46
CCG	3	68	55	0.26	5,940	0.05	564	87.35	140.92	29.12	0.64	0.806
AAAC	4	17	17	0.08	996	0.01	120	58.59	33.20	14.65	0.11	0.20
AAAG	4	89	88	0.42	7,128	0.06	249	80.09	31.49	20.02	0.77	1.05
AAAT	4	95	93	0.44	7,572	0.07	372	79.71	60.36	19.93	0.82	1.13
AAGC	4	9	9	0.04	707	0.01	348	78.56	101.09	19.64	0.08	0.11
AAGG	4	33	31	0.15	4,890	0.04	642	148.18	169.76	37.05	0.53	0.39
AATG	4	11	11	0.05	923	0.01	131	83.91	31.85	20.98	0.10	0.13
ACAG	4	46	46	0.22	7,787	0.07	639	169.28	155.00	42.32	0.85	0.55
ACAT	4	82	79	0.38	10,505	0.10	659	128.11	133.48	32.03	1.14	0.97
ACGC	4	18	18	0.09	1,250	0.01	140	69.44	33.17	17.36	0.14	0.21
ACGG	4	89	89	0.43	3,789	0.03	116	42.57	9.40	10.64	0.41	1.05
ACTC	4	34	34	0.16	5,470	0.05	727	160.88	157.78	40.22	0.59	0.40
AGAT	4	76	75	0.36	10,076	0.09	743	132.58	114.86	33.14	1.09	0.90
AGCC	4	134	134	0.64	17,294	0.16	330	129.06	43.55	32.26	1.88	1.59
AGCG	4	63	62	0.30	2,054	0.02	36	32.60	2.82	8.15	0.22	0.75
AGGC	4	62	62	0.30	5,970	0.05	415	96.29	71.97	24.07	0.65	0.74
AGGG	4	97	94	0.45	13,231	0.12	618	136.40	130.46	34.10	1.44	1.15
ATCC	4	6	6	0.03	432	0.00	170	72.00	57.96	18.00	0.05	0.07
CCCG	4	29	29	0.14	1,915	0.02	82	66.03	8.26	16.51	0.21	0.34
AAAAC	5	6	6	0.03	388	0.00	96	64.67	24.05	12.93	0.04	0.07
AAAAG	5	14	14	0.07	927	0.01	115	66.21	28.21	13.24	0.10	0.17
AAAAT	5	9	9	0.04	435	0.00	60	48.33	10.05	9.67	0.05	0.11
AACCT	5	131	126	0.60	41,414	0.37	705	316.14	205.30	63.23	4.49	1.55
AAGAG	5	24	24	0.11	3,946	0.04	676	164.42	167.47	32.88	0.43	0.28
AAGGG	5	12	12	0.06	1,930	0.02	414	160.83	143.66	32.17	0.21	0.14
AATAT	5	14	14	0.07	1,236	0.01	258	88.29	58.22	17.66	0.13	0.17
AGAGG	5	16	15	0.07	1,873	0.02	456	117.06	116.10	23.41	0.20	0.19
AGGCG	5	25	25	0.12	922	0.01	37	36.88	0.60	7.38	0.10	0.30
AGGGG	5	20	20	0.10	1,884	0.02	215	94.20	53.84	18.84	0.20	0.24
AAAAAG	6	13	13	0.06	1,703	0.02	437	131.00	119.28	21.83	0.19	0.15
AAAAAT	6	9	9	0.04	452	0.00	62	50.22	16.36	8.37	0.05	0.11
AAACAC	6	6	6	0.03	425	0.00	133	70.83	32.20	11.81	0.05	0.07
AAAGAG	6	26	26	0.12	4,633	0.04	705	178.19	156.46	29.70	0.50	0.31
AAATAT	6	6	4	0.02	184	0.00	59	30.67	16.68	5.11	0.02	0.07
AAATGG	6	6	3	0.01	218	0.00	79	36.33	23.55	6.06	0.02	0.07
AACAAT	6	7	7	0.03	625	0.01	119	89.29	22.26	14.88	0.07	0.08
AACAGC	6	25	25	0.12	1,052	0.01	55	42.08	5.61	7.01	0.11	0.30
AAGAGC	6	8	8	0.04	352	0.00	44	44.00	0.00	7.33	0.04	0.10
AAGAGG	6	30	30	0.14	4,223	0.04	554	140.77	124.39	23.46	0.46	0.36
AAGGAG	6	28	28	0.13	3,377	0.03	318	120.61	85.98	20.10	0.37	0.33
AAGGGG	6	23	23	0.11	2,123	0.02	195	92.30	55.98	15.38	0.23	0.27
AATATT	6	7	7	0.03	370	0.00	98	52.86	30.56	8.81	0.04	0.08
AATGAT	6	45	41	0.20	5,325	0.05	453	118.33	107.96	19.72	0.58	0.53
ACACAT	6	16	16	0.08	2,716	0.02	714	169.75	190.21	28.29	0.30	0.19
ACACGC	6	14	14	0.07	1,056	0.01	136	75.43	32.56	12.57	0.12	0.17
ACACTC	6	10	10	0.05	972	0.01	172	97.20	46.96	16.20	0.11	0.12
ACAGAG	6	13	13	0.06	2,486	0.02	733	191.23	196.79	31.87	0.27	0.15
ACATAT	6	15	13	0.06	1,737	0.02	367	115.80	104.02	19.30	0.19	0.18
ACCATC	6	11	11	0.05	852	0.01	203	77.45	66.27	12.91	0.09	0.13
ACCTCC	6	8	8	0.04	587	0.01	150	73.38	50.83	12.23	0.06	0.10
ACGATG	6	9	9	0.04	424	0.00	86	47.11	22.13	7.85	0.05	0.11
ACTCTC	6	6	6	0.03	752	0.01	229	125.33	84.80	20.89	0.08	0.07
AGAGGG	6	49	49	0.23	7,856	0.07	702	160.33	138.90	26.72	0.85	0.58
AGCCGC	6	10	10	0.05	642	0.01	175	64.20	43.00	10.70	0.07	0.12
AGGATG	6	6	6	0.03	929	0.01	509	154.83	181.98	25.81	0.10	0.07
AGGGGG	6	19	19	0.09	2,064	0.02	321	108.63	73.56	18.11	0.22	0.23
CCCCCG	6	66	25	0.12	838	0.01	58	12.70	5.66	2.12	0.09	0.78

*^a ^*The characterization of microsatellites in *P. monodon *FESs is accomplished in terms of the occurring hits*^b^*, the number of occurred FESs*^c^*, the percentage frequency of repeats in terms of the total number of FES analyzed*^d^*, the amount of repeats in terms of nucleotide length*^e ^*and as a percentage*^f ^*of total sequence (11,114,786 bp) analyzed, the maximum length*^g^*, the standard deviation*^i ^*about the microsatellite mean length*^h^*, the mean repeat number*^j^*, the relative abundance*^k^*, and the relative frequency*^l^*. In total, seventy-one microsatellite types have ≧6 hits in the 20,926 FESs; the remaining 91 microsatellite types with 1~5 hits were omitted.

*^k^*The relative abundance (RA) is the base-pairs comprised by each repeat class [Bases (bp)***^e^***] divided by the total length of all microsatellites (921,720 bp) ×100.

*^l ^*The relative frequency (RF) is the hits of each repeat class [Counts*^b^*] divided by the total hits of all microsatellites (8,441 hits) ×100.

In term of repeat motif length, among all microsatellite classes, dinucleotide repeats have the highest relative frequency and relative abundance (42.4%, 44.3%), followed by trinucleotide (33.0%, 31.0%), tetranucleotide (11.9%, 11.3%), hexa-nucleotide (7.1%, 6.2%), penta-nucleotide (3.7%, 6.3%) and mononucleotide repeats (1.9%, 0.9%).

Among dinucleotide repeats, AG repeats are the most abundant with relative frequency of 16.9% and relative abundance of 21.5%, followed by AC (13.7%, 12.8%) and AT repeats (10.8%, 9.5%). CG repeats are present in low relative frequency (1.1%) and relative abundance (0.4%), as observed in other invertebrates, mammals and plants, probably due to the structural problems it may have on DNA conformation [8]. Among trinucleotide repeats, AAT repeats, with relative frequency of 10.8% and relative abundance of 14.2%, comprised nearly one half (46%) of the trinucleotide repeats in lengths and were the most abundant in this class, far more than ATC repeats with the second highest relative frequency (6.5%) and abundance (5.4%).

It is noteworthy that one pentanucleotide repeat, AACCT, was particularly abundant compared to all other penta-, tetra-, or even hexa-nucleotide repeats (Table 2). With a relative frequency of 1.6% and a relative abundance of 4.5%, AACCT repeats were the 12^th ^most frequent microsatellite type. Their mean length per locus (316.1 bp) was the highest among the 20 most frequently occurring microsatellite classes. In particular, ~70% of the AACCT repeats are perfect or nearly perfect repeats. The (AACCT/TTAGG) repeat turns out to be the telomere motif in arthropods, which is only 1 base pair different from the ancestral telomere repeat motif (AACCCT/TTAGGG) found in vertebrates and in all other basal metazoan groups [9].

The microsatellite abundance lies on both the distribution frequencies and the sizes of the repeats. When comparing to other taxa, we found that microsatellite sequences in the *P. monodon *genome occur at higher density (Table 3) than in vertebrates. Approximately 1 microsatellite was present in every 1.32 kb, which is 4.6 times more frequent than the one per 6 kb estimated for humans [10]. The frequency of microsatellite sequences in this species was even higher than that in the *Fugu *genome, which have the highest microsatellite density (1 per 1.88 kb) known so far [11].

**Table 3:** Survey of microsatellite distribution and mean lengths in various genomes

Species	% genome (bp)	Density	Mean length (bp)	References
*P. monodon*	8.30	1 per 1.32 kb	109.2	This study
*B. mori*	0.31	1 per 9.56 kb	29.6	[24]
Arthropoda*^a^*	0.54	--	--	[13]
*Fugu*	1.30	1 per 1.876 kb	25.6	[11,12]
Human	1.0-3.0	1 per 6 kb	19.0	[10]

*^a ^*Data mostly from *Drosophila melanogaster *and other 159 *Drosophila *sp.

**Figure 2:**
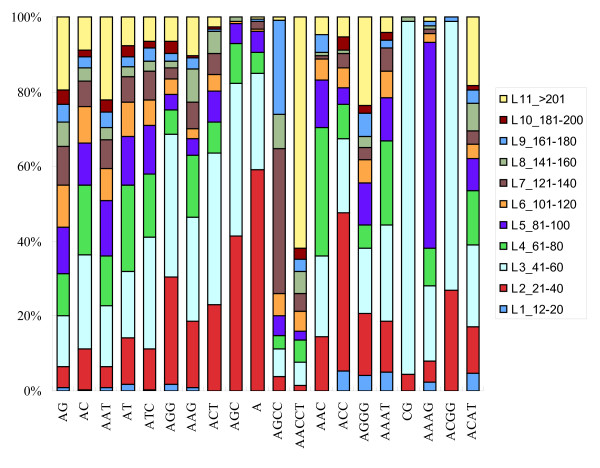
**Length distribution of the 20 most frequently occurring microsatellite classes**. L1 type: 12-20 bp; L2: 21-40 bp; L3: 41-60 bp; L4: 61-80 bp; L5: 81-100 bp; L6: 101-120 bp; L7: 121-140 bp; L8: 141-160 bp; L9: 161-180 bp; L10: 181-200 bp; L11: > 201 bp.

As to the sizes of the repeats, the mean length for individual microsatellite loci in *P. monodon *was unusually long, average 109.2 bp, which is 4 times the 25.6 bp in the *Fugu *genome [12] (Table 3). Of all 8,441 microsatellite loci identified, 84.1% had lengths over 40 bp, and 36.9% had lengths over 100 bp. A total of 135 microsatellite loci had lengths over 500 bp, mostly belonging to AACCT (31 hits; maximal length: 705 bp), AAT (27 hits; 729 bp), AG (22 hits; 763 bp), AC (12 hits; 783 bp), and AAG repeats (8 hits; 720 bp) (Table 2). The longest uninterrupted array of microsatellites was a (TC/AG) repeat, spanning 440 bp with 220 repeat units. Very long stretches of microsatellites in a single read, containing up to six microsatellite loci, were commonly observed. The characteristic of long stretches was also revealed in the length distribution of the 20 most frequently occurring microsatellites classes (Figure 2). Almost each class, except A repeats, had over one half of the loci with lengths exceeding 40 bp, and most of them had over 20% of the loci with lengths exceeding 100 bp. The high frequency and the long lengths of microsatellites in the *P. monodon *genome lead to a decreased sequencing success rate (down to ~70% from a typical rate of ~90%).

With a few exceptions, the length distribution patterns within each of these microsatellite classes are generally consistent (Figure 2). In particular, AACCT repeats had a notably high ratio of longer stretch, with 61.8% of the loci having lengths over 201 bp. In general, the microsatellites with a higher GC% tend to have shorter lengths. For example, CG repeats had a very narrow length range around 42-46 bp (94.5% at L3 type), and ACGG repeats had 98.9% of the loci with the lengths of 21-60 bp (the L2 to L3 types). The only exception is AGCC repeats; despite of the same GC% (75%) as those of ACGG repeats, most of the loci (73.9%) identified had longer lengths more than 120 bp.

High abundance of microsatellite sequences were also found in the transcribed regions. By examining the amount and distribution of microsatellites in one *P. monodon *EST dataset (PmTwN), repeat motifs were found in 8.1% of the uniquely expressed sequences, covering 1.12% of the EST lengths (11,161 bp per Mb). In comparison with other taxa that have been surveyed such as primates (1,515 bp per Mb) and rodents (2,488 bp per Mb) [13], the fraction of microsatellites in the expressed sequences in *P. monodon *is apparently higher. Of all microsatellite classes present in the expressed sequences, dinucleotide- and trinucleotide-repeats were predominant. AT-rich microsatellite types, especially poly (AT) and poly (AAT), were the most abundant, consistent with the result obtained by Maneeruttanarungroj *et al*. [14].

**Figure 3:**
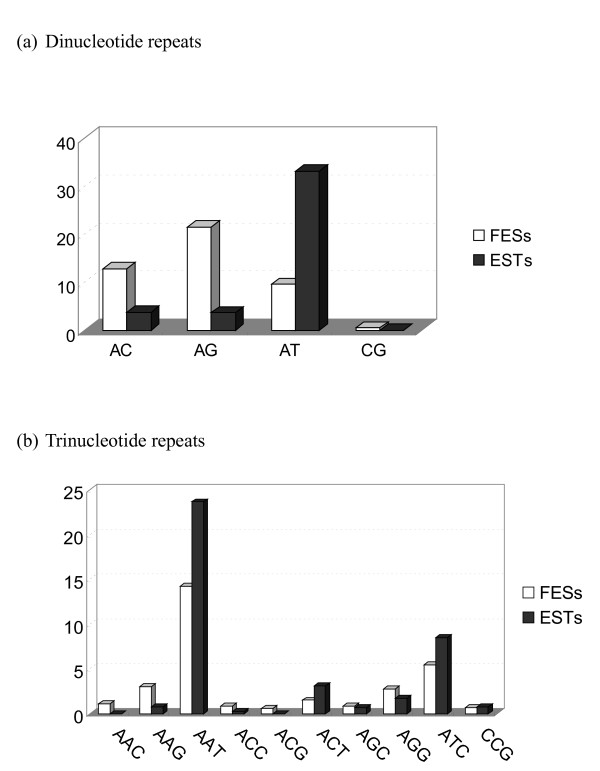
**Comparison between the relative abundance (%) of dinucleotide repeats**. (a) and trinucleotide repeats (b) in genomic DNAs (FESs) and in transcribed regions (ESTs).

In contrast to those in genome average, the frequency distribution of microsatellites with AT-rich motifs [*e.g*., (AT)_n _and (AAT)_n_] in transcribed regions were apparently different (Figure 3), suggesting that different selective and/or mutational pressures are operating on coding and on other genomic regions. In addition, many EST contigs (Additional file 4a) and a number of known genes (Table 4) contained one or multiple microsatellites with notably long string of perfect repeats, most of which were dinucleotide repeats. For example, the *P. monodon *Anti-Virus (*PmAV*) gene (GB# DQ641258) [15] is known to contain a 280 bp-compound imperfect microsatellite repeat [(GT)_46_] within its 5'-promoter region (Table 4). Moreover, at least some of the long microsatellites located within genes showed copy number variation, as demonstrated in several sets of ESTs apparently transcribed from the same gene (Additional file 4b).

**Table 4:** Examples of shrimp genes known to contain a very long stretch of microsatellites

Gene [species]	Gene function	Sequence	Position	Role of microsatellites	Reference
*PmAV *[*P. monodon*]	Anti-virus	**(GT)_46_**[(AT)(GT)_2_]_7_....20 bp.....(AT)_2_(GT)_2_(AT)_3_(GT)_3_(AT)_3_**(GT)_50_**	5'-promoter	Negative regulatory element	[15]
Prophenoloxidase-*a *[*P. vannamei*]	Innate immunity	**(CT)_20_**	3'UTR	n.d.	[32]
Prophenoloxidase-*b *[*P. vannamei*]	Innate immunity	**(CT)_38_**...12 bp...**(CA)_14_**	3'UTR	n.d.	[33]
Heat shock cognate 70 [*P. monodon*]	Molecular chaperon; immune and stress-related responses	**(TA)_33_**...73 bp...(CA)_9_(TG)_2_**(TA)_17_**	5'-promoter	n.d.	[66]
5-HT1 receptor [*P. monodon*]	Serotonin receptor; G-protein coupled	**(GGC)_10_**	Coding region	poly(G) tract	[67]

n.d.: not determined

### Novel repetitive elements

Our data above indicates an apparently lower fraction of transposable elements (less than 1%; Table 1) in the tiger shrimp, in comparison to 45% in the human genome [16] and 16% in the *A. gambiae *genome [17]. We therefore suspected that a large number of specific repeat types could not be detected using the existent repeat database. To unravel novel repetitive elements in *P. monodon*, we used the RECON program [18] to perform all *versus *all BlastN search in the 20,926 repeat-masked FESs. After filtering and collapsing families (see Methods), we identified 103 penaeid repetitive element (PRE) families (Table 5), with a total length of 4,867,916 bp comprising 43.8% of the *P. monodon *genome.

**Table 5:** Summary of the 103 novel repetitive elements in the *P.monodon *genome

(i) WSSV-related (33 PREs)									
**PRE***^b^*	**Counts**	**Consensus (kb)**	**GC%**	**Total length (kb)**	**# matched ESTs*^c^***	**Tandem repeats within**	**Putative gene [Species]***^d^*	**E value**	**Identity**

FAM9_15-44	470	24.000	45.88	274.988	4	21 bp-repeat ×19.4, 45 bp-repeat ×14.0, 66 bp-repeat ×6.5, 24 bp-repeat ×9.5, 24 bp-repeat ×3.1	wsv134 [WSSV]	3.0E-13	31% (44/140)
							wsv115 [WSSV]	5.0E-126	30% (266/876)
							wsv119 [WSSV]	4.0E-25	22% (162/717)
							wsv216 [WSSV]	1.0E-41	23% (291/1261)
							wsv220 [WSSV]	1.0E-37	23% (178/747)
							Hypothetical protein [*Trypanosoma brucei*]	5.0E-19	34% (103/297)
FAM152	461	22.978	44.47	266.056	4	93 bp-repeat ×2.0	wsv447 [WSSV]	3.0E-138	28% (415/1434)
							wsv332 [WSSV]	2.0E-59	25% (212/820)
							wsv327 [WSSV]	2.0E-38	24%(217/902)
							wsv282 [WSSV]	7.0E-63	43% (168/389)
							wsv285 [WSSV]	3.0E-38	22% (186/829)
FAM2	434	19.895	47.82	255.227	4	97 bp-repeat ×2.0, 170 bp-repeat ×2.4	wsv360 [WSSV]	0	30% (899/2911)
FAM1	413	17.987	40.84	237.935	3	60 bp-repeat ×2.1; 185 bp-repeat ×2.5	wsv306 [WSSV]	1.0E-50	32% (121/376)
							wsv269 [WSSV]	2.0E-31	25% (99/388)
FAM31&207	364	24.078	44.71	206.653	4	333 bp-repeat ×1.9, 72 bp-repeat ×2.0, 18 bp-repeat ×7.0, 66 bp-repeat ×2.0, 21 bp-repeat ×2.0	wsv343 [WSSV]	2.0E-81	28% (187/660)
							Inhibitor of Apoptosis Protein [*P. monodon*]	1.0E-115	61% (207/337)
							Innexin 3 [*Acyrthosiphon pisum*]	3.0E-55	40% (104/258)
FAM87	242	12.067	43.76	139.605	4		wsv514 [WSSV]	0	37% (702/1881)
FAM24	209	9.553	47.93	112.279	2		wsv192 [WSSV]	2.0E-108	29% (310/1042)
							wsv209 [WSSV]	0	36% (595/1629)
FAM5	200	8.457	46.61	113.975	1		wsv440 [WSSV]	2.0E-54	28% (180/627)
							wsv433 [WSSV]	6.0E-163	39% (365/928)
							wsv427 [WSSV]	3.0E-20	27%(91/332)
FAM28	131	6.481	43.94	74.195	0		wsv325 [WSSV]	6.0E-60	33% (165/493)
							wsv271 [WSSV]	5.0E-13	19% (120/608)
FAM43	127	7.919	47.99	70.954	2		wsv011 [WSSV]	3.0E-86	27% (332/1190)
FAM255	119	8.153	41.42	66.930	0		wsv514 [WSSV]	1.0E-77	27% (294/1071)
FAM124	116	4.961	48.90	63.455	0		wsv026 [WSSV]	4.0E-90	39% (231/592)
FAM179	88	4.819	52.25	51.657	0		wsv035 [WSSV]	1.0E-134	35% (221/616)
							wsv037 [WSSV]	1.0E-94	35% (204/581)
FAM361*	84	5.845	43.92	47.611	2		wsv209 [WSSV]	3.0E-80	30% (213/697)
FAM259*	82	7.353	42.46	45.564	7		wsv306 [WSSV]	1.0E-33	27% (119/427)
							wsv332 [WSSV]	2.0E-12	22% (82/362)
FAM137	71	2.639	43.08	39.233	2		wsv303 [WSSV]	4.0E-97	32% (266/819)
FAM158	68	3.912	49.85	34.857	2		wsv289 [WSSV]	2.0E-19	22% (144/634)
FAM29	67	2.661	43.22	35.159	0		wsv423 [WSSV]	4.0E-81	32% (195/594)
FAM197	52	3.181	44.39	27.452	0		wsv289 [WSSV]	1.0E-20	31% (59/188)
FAM483	41	3.624	44.40	21.374	0		wsv360 [WSSV]	7.0E-30	25% (129/511)
FAM224&1875	41	2.462	42.12	19.675	0		wsv360 [WSSV]	4.0E-40	32% (119/368)
FAM411	41	1.739	49.17	22.235	0		wsv037 [WSSV]	1.0E-56	35% (151/425)
FAM541	38	3.175	37.76	21.559	0		wsv343 [WSSV]	4.0E-36	25% (126/490)
FAM209	34	3.429	46.60	18.964	0		wsv035 [WSSV]	1.0E-95	31% (309/986)
FAM346	34	3.115	44.37	18.965	0		wsv011 [WSSV]	1.0E-42	32% (113/345),
FAM138	33	2.153	36.79	16.305	0		wsv433 [WSSV]	4.0E-71	31% (205/655)
FAM177	31	2.105	37.62	13.970	0		wsv433 [WSSV]	2.0E-41	31% (147/472)
FAM56	26	4.241	39.85	14.764	0	15 bp-repeat ×2.5, 18 bp-repeat ×2.2	wsv115 [WSSV]	2.0E-25	29% (117/402)
FAM472	26	1.930	45.70	13.534	0		wsv447 [WSSV]	6.0E-42	25% (153/610)
FAM156_3,4	26	1.752	41.44	14.790	0		wsv139 [WSSV]	1.0E-38	30% (120/388)
FAM838	26	1.616	41.89	11.572	0		wsv360 [WSSV]	4.0E-52	31% (169/538)
FAM574	25	3.916	40.70	14.908	0		wsv026 [WSSV]	6.0E-50	34% (178/522)
FAM139	24	2.431	40.93	13.449	2		wsv360 [WSSV]	2.0E-14	22% (68/297)

**Total**				**2,399.849 (21.6%)**					

**(ii) Retrotransposon-related (14 PREs)**									

**PRE***^b^*	**Counts**	**Consensus (kb)**	**GC%**	**Total length (kb)**	**#matched ESTs*^c^***	**Tandem repeats within**	**Type*^e^***	**E value**	**Note**

FAM9_1-14	392	6.853	43.02	190.147	69	177 bp-repeat ×1.9	LINE/I	2.6E-144	Including a previously described retrotransposon (Contig T; GB# EE724330) demonstrated to be down-regulated under hypoxic and hyperthermic stress [28]
FAM185	350	5.837	40.86	280.277	32		LINE/I	3.8E-51	Including a sex-linked AFLP marker (E03M60M72.8) [19], and a previously described retrotransposon (ED 363; GB# EE724313) demonstrated to be down-regulated under osmotic stress [21]
FAM309	222	7.612	40.95	126.973	1		Penelope	4.1E-40	
FAM189_7-16	201	6.250	44.43	112.035	2		Penelope	1.3E-43	
FAM75_17-25, 35-36,39-40	163	4.207	50.8	82.963	11		LINE/Jockey	4.2E-46	Including a previously described retrotransposon (Contig X; GB# EE724334) which is differentially expressed under various stress [21].
FAM18*	143	4.621	36.57	76.777	1		Penelope	1.3E-47	
FAM75_1-9, 11-16	118	4.148	57.11	51.196	23	30 bp-repeat ×2.1	LTR/Gypsy	1.8E-25	
FAM9_45-54	109	4.024	52.81	45.069	41		LINE/RTE-BovB	1.4E-92	Including 2 previously described retrotransposons- (1) GB# DQ228358 [20], and (2) GB# EE724273 (Contig AK) which is up-regulated under osmotic and hyperthermic stress [21].
FAM189_1-6	64	5.041	43.42	34.129	0	16 bp-repeat ×2.1	Penelope	2.0E-41	
FAM380	58	3.330	40.33	28.528	0	30 bp-repeat ×2.5	Penelope	1.2E-39	
FAM393	52	3.321	44.11	28.283	19		LTR/Gypsy	1.7E-14	
FAM1285	21	2.233	40.17	10.649	0		LINE/Jockey	1.2E-30	
FAM498	26	1.928	48.34	10.537	0		LINE/Jockey	4.0E-10	Containing a *PmAV*-like sequence
FAM1106	24	0.828	38.29	7.719	12		LINE/RTE-BovB	2.7E-30	Including a previously described retrotransposon (ED255; GB# EE724266) which is up-regulated under hyperthermic stress [21].

**Total**				**1,085.282 (9.8%)**					

**(iii) Annotated (5 PREs)**									

**PRE***^b^*	**Counts**	**Consensus (kb)**	**GC%**	**Total length (kb)**	**# matched ESTs*^c^***	**Tandem repeats within**	**Putative gene [Species]***^d^*	**E value**	**Identity**

FAM145*	50	3.792	37.61	29.848	0	21 bp-repeat ×2.0, 26 bp-repeat ×3.0	dUTPase isoform 1 [*Apis mellifera*]	5.0E-53	69% (96/139)
FAM327	54	6.411	37.65	26.625	1	29 bp-repeat ×1.9	Heat Shock Protein 70 [*Homarus americanus*]	4.0E-71	75% (136/180)
FAM142	31	2.598	42.73	17.365	0	40 bp-repeat ×2.3	Heat Shock Protein 70 [*Demania scaberrima*]	2.0E-125	56% (251/441)
FAM46*	26	2.712	37.21	12.028	1		Inhibitor of Apoptosis Protein [*P. monodon*]	9.0E-31	34% (100/294)
FAM575	35	1.351	42.78	11.594	2	48 bp-repeat ×11.4 48 bp-repeat ×3.9	hCG1645741 [*Homo sapiens*]	3.0E-15	30% (100/328)

**Total**				**97.460 (0.9%)**					

**(iv) Unannotated (51 PREs)**									

**PRE***^b^*	**Counts**	**Consensus (kb)**	**GC%**	**Total length (kb)**	**# matched ESTs*^c^***	**Tandem repeats within**			

FAM42	419	0.611	54.83	168.921	0	15 bp-repeat ×2.0			
FAM72	309	1.306	61.72	130.598	2				
FAM121	153	1.368	56.07	90.081	0	472 bp-repeat ×2.3			
FAM80	158	0.618	73.79	56.266	0				
FAM75_26-34, 37-38	88	2.565	53.33	43.871	0	190 bp-repeat ×2.6; (GGAGAGAGGGGA) ×2.3			
FAM198	84	7.608	39.34	47.826	0	192 bp-repeat ×1.8			
FAM205	83	0.400	49.00	16.051	0				
FAM345	80	0.284	47.89	17.102	0	(GTGTTGGTTTGTGT) ×2.2			
FAM67&606&707	75	3.578	42.51	38.079	3	24 bp-repeat ×2.0; 273 bp-repeat ×1.9; 15 bp-repeat ×2.0			
FAM328	66	2.892	39.42	37.000	0	21 bp-repeat ×2.1			
FAM79	63	0.123	73.17	7.475	0				
FAM19	55	4.948	47.15	32.320	0	13 bp-repeat ×3.1			
FAM6	57	2.133	46.51	28.644	0				
FAM64*	57	2.958	39.69	27.477	0				
FAM199	51	2.362	41.19	24.646	0				
FAM156_1,2,5	50	2.180	43.58	26.492	0				
FAM392	50	0.904	31.42	14.760	0				
FAM188	49	0.990	48.59	12.318	17	302 bp-repeat ×1.9			
FAM348	46	0.244	72.54	9.579	0				
FAM165	45	0.944	34.32	25.341	0	129 bp-repeat ×6.7			
FAM382*	44	1.995	41.60	24.050	0				
FAM153	36	1.895	42.90	16.403	0				
FAM245	36	1.626	47.72	17.019	0				
FAM578	35	3.552	45.02	20.560	0				
FAM57	33	3.366	39.51	18.344	4				
FAM453	33	0.802	43.14	13.902	0	59 bp-repeat ×2.0			
FAM172	32	4.035	40.57	18.414	0				
FAM632	32	3.077	38.12	17.055	1				
FAM839	32	1.896	30.96	20.315	2	18 bp-repeat ×1.9; 61 bp-repeat ×2.1; 215 bp-repeat ×2.1			
FAM120	31	2.782	38.75	18.864	0	(GCCTGA) ×5.8, (CTGCGG) ×4.2			
FAM390	31	0.563	38.54	11.570	0				
FAM369	30	1.579	41.36	15.870	0				
FAM816	27	3.056	36.81	15.590	0	87 bp-repeat ×6.7			
FAM22	27	3.767	42.39	14.516	0	39 bp-repeat ×2.2			
FAM708	26	3.297	37.03	17.053	0	37 bp-repeat ×2.1; 34 bp-repeat ×1.9; 17 bp-repeat ×1.9 × 2 sites			
FAM244	25	1.269	39.48	11.190	2				
FAM405	25	0.963	42.37	11.828	0				
FAM580	24	2.246	37.62	13.474	0	183 bp-repeat ×3.1; 34 bp-repeat ×1.9			
FAM266	24	0.486	51.23	5.216	0				
FAM440	23	2.100	39.71	12.938	0	27 bp-repeat ×2.9; 162 bp-repeat ×2.6			
FAM1203	23	1.298	41.68	11.308	0				
FAM1696	23	0.495	37.37	7.108	0				
FAM569	22	2.189	39.10	11.903	3				
FAM296	22	2.169	39.47	11.409	0				
FAM1318	21	2.597	34.39	11.053	0				
FAM146	21	2.557	44.66	12.539	0				
FAM278	21	2.324	38.60	11.618	0				
FAM282	21	1.875	48.05	10.369	0				
FAM277	21	1.705	36.60	9.807	0				
FAM740	20	1.293	31.01	7.472	0				
FAM696	20	0.755	29.27	11.730	1	86 bp-repeat ×8.6			

**Total**				**1,285.334**	**(11.6%)**				

*^a^*The consensus sequences of the 103 PREs can be downloaded from the Penaeus Genome Database http://sysbio.iis.sinica.edu.tw/page/others.php?news=0.

*^b^*Seven out of the 103 PREs of which homologous sequences were present in the *Marsupenaeus japonicus *genome were marked with asterisks (*).

*^c^*BlastN search against the *P. monodon *EST dataset (PmTwN) in the Penaeus Genome Database; cut-off value: 1E-40; matched length: 200 bp; identity: 85%

*^d ^*BlastX search against the *nr *database; cut-off value: 1E-10

*^e^*Types of transposable element defined by protein-based RepeatMasker; cut-off value: 1E-10

The 103 PREs can be categorized into four groups according to the similarity to sequences in the public databases (Table 5). The first group, comprising an estimated 21.6% of the *P. monodon *genome and containing 33 PREs, showed only moderate similarity (19%-55% identities) to a number of white spot syndrome virus (WSSV) genes, suggesting these WSSV-like sequences are part of the shrimp genome rather than from complete virions. WSSV is one of the most deadly viruses that have plagued the shrimp farming industry. These WSSV-like sequences probably are the proviral remnants of ancestral germ-line infections by active WSSVs, degenerating to an extent that they lost their functional potential as a virus. Most of the very long repeats were contained in this group, in some of which even more than one WSSV-related sequences were found. The PRE with the longest consensus was FAM31&207 (24.08 kb). This PRE not only contained one wsv343-related segment, but also two other non-overlapping regions similar to Inhibitor of Apoptosis Protein (IAP) gene and Innexin-3 gene. The PRE with the highest count number was FAM9_15-44 (470 hits in the 20,926 FESs), which contained at least five WSSV-related sequences.

The second group, comprising an estimated 9.8% of the *P. monodon *genome and containing 14 PREs, showed similarities to transposon-related sequences such as *pol*, *gag*, and reverse transcriptase genes. These 14 PREs, not well-represented in the Repbase (Repetitive DNA database), were older/more divergent members of the transposable element families and can only be annotated by protein-based RepeatMasker. All of the 14 novel transposons were retrotransposons. Seven of them belong to non-LTR retrotransposons, also known as LINEs (Long Interspersed Nuclear Elements), representing 3 (RTE, I, and Jockey) of the 15 previously described clades. An additional five belong to a unique class of retrotransposons called Penelope. And only two were LTR retrotransposons, both belonging to the *gypsy *clade. The PRE with the longest consensus was FAM309 (7.61 kb), being a Penelope element. The PRE with the highest number abundance was FAM9_1-14 (392 hits in the 20,926 FESs), belonging to the LINE/I clade. The PRE with the highest combined length was FAM185 (280 kb), also belonging to the LINE/I clade. It is noteworthy that a 60-bp sex-linked AFLP marker (E03M60M72.8), previously demonstrated as a female-associated *W *allele by linkage analysis and further confirmed on a population scale [19], apparently was derived from a member of FAM185. In addition, FAM498 is notable for carrying a *PmAV*-like sequence (nucleotide similarity = 95% [1658/1761]; E value: 0), indicating that a *PmAV *gene/sequence was transposed by a non-LTR retrotransposon. Some previously identified retrotransposons were verified and included in this group. For instance, a retrotransposon (GB# DQ228358) which was originally discovered due to its proximity to an IHHNV-related sequence in the African and Australian *P. monodon *genomes [20] falls in the repetitive element family FAM9_45-54. And several retrotransposons showing differential expression in response to a range of environmental stressors [21,22] were demonstrated as members of 4 PREs, *i.e*., FAM9_1-14, FAM185, FAM75_17-25,35-36,39-40, and FAM9_45-54.

The third group, comprising 0.9% of the *P. monodon *genome and containing 5 PREs, matched known genes with a minimum length of 100 bp (equivalent to 34 amino acids) and with a minimum identity of 30% (30%~75%). This group might include large gene families with a great number of duplicated genes and pseudogenes, such as Heat Shock Protein 70 gene (FAM327 and FAM142) and Inhibitor of Apoptosis Protein gene (FAM31&207 and FAM46).

The fourth group, comprising an estimated 11.6% of the *P. monodon *genome and containing 51 PREs, did not match any known sequences. Some of them, *e.g*., FAM165, FAM816, and FAM696, contained minisatellites. Twenty PREs had the consensus shorter than 1.5 kb, four of which had a high GC content > 60%. The PRE with the longest consensus was FAM198 (7.61 kb). The PRE with the highest count number was FAM42 (419 hits in the 20,926 FESs), resulting in an extraordinary total length of ~169 kb despite of only 611 bp per repeat sequence.

To determine if the repetitive element families identified are transcriptionally active, their consensus sequences were searched for similarity in the Penaeus Genome Database http://sysbio.iis.sinica.edu.tw/page/[6], which includes over 200,000 Expressed Sequence Tags (ESTs) from four penaeid shrimps. Of the 103 repetitive element families, thirty-six had significant hits to *P. monodon *ESTs, implying expression of their portions in some members of the repetitive family as transcripts (Table 5 Additional file 5). These 36 transcriptionally active PREs included 14 WSSV-derived PREs (FAM31&207 was categorized into this group), 10 retrotransposons, 3 gene family-like PREs, and 9 unannotated PREs. Moreover, we found evidence indicating that some of the WSSV-like and the reverse transcriptase-like segments are active as transcribed RNAs. In 9 of the 14 transcriptionally active WSSV-derived PREs, transcripts derived from the WSSV-related region were found. And in 7 of the 10 transcriptionally active retrotransposon-derived PREs, the reverse transcriptase- or *pol*-like sequences seem to be expressed. However, among the 3 PREs assumed to be gene families, only one PRE (FAM575) seems to derive expression exactly from their putative protein-coding region.

In summary, the fact that a variety of repeats comprise a significant fraction of the *P. monodon *genome highlights its highly repetitive nature. If all repetitive elements identified both by RepeatMasker and RECON were included, up to 79.29% (16,592/20,926) of end sequence reads contain interspersed and/or tandem repeats. In terms of lengths, the repetitive sequences in the *P. monodon *genome comprise 51.18% of the *P. monodon *genome (Table 6). Retrotransposon-derived, WSSV-related sequences, and unknown/unannotated sequences comprised 10.75%, 21.59%, and 11.57% of the genome in length, respectively. These estimates are conservative because more repetitive elements are expected to be identified if the criteria for defining a repetitive element family are less stringent. Moreover, sequences homologous to 7 of the 103 PREs (Table 5) were found to be present in one kuruma shrimp BAC clone (Mj024A04), known to be highly repeated in the *Marsupenaeus japonicus *genome [23]. This suggests that the extreme repetitiveness might be a common feature of penaeid genome.

**Table 6:** Summary of repetitive sequences in the *P.monodon *genome

		Length (bp)		Total length (%)
			
Type		RepeatMasker	RECON	
**Retrotransposon**	Non-LTRs	16,570	627,361	643,931 (5.79%)
	Penelope	0	378,442	378,442 (3.40%)
	LTRs	77,962	79,479	157,441 (1.42%)
**DNA transposon**		14,817	0	14,817 (0.13%)
**WSSV-related PREs**		0	2,399,849	2,399,849 (21.59%)
**Unknown/unannotated PREs**		309	1,285,334	1,285,643 (11.57%)
**Microsatellites**		807,927	0	807,927 (7.27%)

**Total**				5,688,050 **(51.18%)**

### Protein-coding sequences

To identify putative protein-coding sequences and to estimate gene density in the *P. monodon *genome, 20,926 fosmid end sequences were subjected to sequence similarity searches using BlastN on the Penaeus Genome Database. Approximately 28.2% (5,910 FESs) of the 20,926 FESs exhibited 80-100% identity (cut-off value: E-10) to 3,983 shrimp ESTs; however, 59.0% (3,471/5,910) of them were found to be derived from 98 PREs and were excluded. Most of the remaining FESs (57.5% = 1,403/2,439) were related to r-RNA genes; only 590 (2.8%) FESs might contain protein-coding genes after excluding those deriving from mitochondria and transposons. Among them, 399 matched to the ESTs of their own species, and 191 matched to the ESTs of other penaeid species. An alternative approach to identify protein-coding sequences within the FESs is to perform BlastX search on the NCBI *nr *protein database. Approximately 4.8% (994/20,926) of the 20,926 FESs showed sequence similarity to 586 nuclear genes, although most of the genes are unannotated or of unknown function. Notably, two gene homologues, IAPs (41/994) and Innexins (11/994), were shown to be present in high copy numbers in the FESs, suggesting that they are present in high abundance in the *P. monodon *genome. Overall, in combination of the BlastX search against the *nr *database and BlastN in the *P. monodon *EST database, we identified 1,541 (7.4%) FESs containing protein-coding genes. To estimate the gene count in the *P. monodon *genome in a more conservative approach, we used the result of BlastN against the EST database only. Providing that the average gene size ranges from 7 kb (estimated from one *P. monodon *fosmid clone; unpublished data) to 10 kb (as humans), the gene count in the *P. monodon *genome was estimated to be 10,400-14880 [= 4.8%× (2.17×10^6^kb)/gene size], with the gene density of one gene per 145-208 kb. These FESs containing significant hits with coding genes will be important for gene localization and will provide information for defining the gene structure.

## Discussion

### High abundance of microsatellites in the *P. monodon *genome

This is the first large-scale survey on the repeats in the tiger shrimp genome. Here we found that the *P. monodon *genome contains a significant proportion (8.3%) of microsatellite sequences, greater than those of other arthropods such as *Drosophila *species (0.54%) [13] and silk moth *Bombyx mori *(0.31%) [24] (Table 3). It is also much higher than the frequency of ~ 1% in many vertebrates including primates [25], human and rat [10], pig and chicken [26], rabbit [27] and *Fugu *(1.3%) [11]. The presence of large quantities of microsatellite sequences seems to be a distinct characteristic of penaeid genomes, like those of *F. chinensis*[28] and *P. vannamei*[29]. The mechanism that determines and maintains the abundance of tandem repeats is not well understood, but apparently reflects the response of the whole genome to overall selective and mutational pressures [30]. It is also plausible that transposable elements might contribute to the formation and the spread of the highly repetitive satellite DNAs by means of unequal crossing over [31].

Abundant microsatellites were also found in the transcribed regions. Similar results were obtained by Maneeruttanarungroj *et al*. [14], which revealed that 9.9% of the *P. monodon *ESTs (997/10,100) contained microsatellites. In addition, by reviewing the literature (Table 4) and by examining the *P. monodon *EST dataset in the Penaeus Genome Database (Additional file 4a), we found that many shrimp genes/ESTs contain long stretches of microsatellites. As longer repeats generally have higher mutation rates, the abundance and long stretches of microsatellites in transcribed regions are unusual, raising the possibility that they may have functional roles. In addition, most of these microsatellites were dinucleotide repeats (Table 4 Additional file 4), implying they act as regulatory elements within the 5'- or 3'-untranslated regions (UTRs) rather than as coding sequences of genes. Otherwise their copy number variation will result in frame-shift mutations. For example, the *PmAV *gene, an antiviral gene which is up-regulated upon viral infection, is known to contain a dinucleotide repeat [(GT)_46_] in the promoter region as a negative regulatory element for *PmAV *expression [15].

Another example is the prophenoloxidase (*proPO*) gene in *P. vannamei *(Table 4). Two forms of *proPO *gene were found, both having a microsatellite near the 3' end of the open reading frame: *proPO-a *(GB# EU373096) has a perfect microsatellite [(CT)_20_] [32], while *proPO-b *(GB# EF115296) has a compound imperfect microsatellite [(CT)_38_(CA)_8_(AA)(CA)_3_(TA)(CA)_14_] [33]. Their 3' end cDNA sequences following this (CT)_n _repeat are different. It has been observed that *proPO-b *expression was down-regulated in the white shrimps challenged with WSSV, but whether this is in any relation to the (CT)_n _repeat remains to be determined.

Microsatellites have been hypothesized to be an important source of quantitative genetic variation and evolutionary adaptation [34-36]. The high mutational rate suggest that microsatellites can act like adjustable tuning knobs through which specific genes are able to rapidly adjust the norm of reaction in response to minor or major shifts in evolutionary demands [37]. In this study, by examining EST database we observed that some microsatellites contained in the genes showed copy number variation, probably representing different alleles (Additional file 4b). One example was a *C*-type lectin-like gene. *C*-type lectin is known to play an important role in innate immunity of invertebrates. Intriguingly, this gene, together with 3 other genes known to have a very long stretch of microsatellites (*PmAV*, *proPO*, and Heat shock cognate 70 gene) (Table 4), are all involved in immune/stress response and possibly undergo frequent regulation of gene expression. This is in agreement with the hypothesis that microsatellites could have a role in adaptive evolution.

### Transposable elements in the *P. monodon *genome

Transposable elements have been shown to occupy a large portion of some eukaryotic genomes, and may have a significant influence on genome evolution [38-40]. They may affect the expression of nearby genes, serve as homologous sites for recombination, and contribute to novel exons [41]. In this study, we identified 14 novel retrotransposons out of the 103 PREs. Together with DNA transposon, transposable elements occupy at least 10% of the *P. monodon *genome. Over one half of the transposable elements in length belong to non-LTR retrotransposons. Five non-LTR retrotransposon clades, CR1, R1, RTE, I, and Jockey clades, were identified (Table 5). Of them, the I clade was apparently the most represented, contributing to 73% (470,424/643,931 bp) of the non-LTR portion of the *P. monodon *genome. One PRE (FAM185) of the I clade was found to include a sex-linked AFLP marker (E03M60M72.8) [19], suggesting that at least one introgression site of non-LTR retrotransposons exists on the sex chromosome, mostly likely the W chromosome of the ZW sex determination system.

The R1 clade is a less represented non-LTR retrotransposon in the *P. monodon *genome. Unlike most other non-LTRs inserting throughout the host genome, however, the R1 clade is known for its distinct target specificity. For example, the R1 clade families RT and R7 have been known to specifically insert in the 28S and 18S ribosomal RNA (rRNA) genes, respectively [42-45]; the Mino elements insert into AC repeats [45]. All of these R1 clade families were found in the *P. monodon *genome, *i.e*., the RT (95 hits), R7 (2 hits), and Mino (2 hits) elements. Consistent with target specificity, a significant portion (4.11%) of the *P. monodon *genome was found to contain highly repetitive short sequences similar to 18S or 28S ribosomal RNA genes, some of which may reflect the remnants of the target-specific retrotransposition of the R1 clade.

Penelope elements are a unique but relatively little studied class of retrotransposons. This type of retrotransposon has been known to insert randomly throughout the genome, preferring AT-rich targets [46]. Penelope elements are also known for their patchy distribution in various taxonomic groups, *e.g*., they are present in only *D. virilis *and *D. willistoni *in a dozen sequenced *Drosophila *genomes, suggesting that they are frequently lost from relatively close species [46,47]. In addition to one Penelope element previously identified, we further found 5 PREs representing Penelope elements, comprising a significant fraction (32.1% = 378.442/1179.814 kb) of the retrotransposon portion of the *P. monodon *genome.

As mentioned above, five previously established non-LTR retrotransposon clades (CR1, R1, RTE, I, and Jockey) have been identified in the *P. monodon *genome (Table 5). Among them, four clades (CR1, R1, I, and Jockey) are commonly found in most of the major arthropod lineages, *e.g*., insects [48], crustaceans, and chelicerates [49], suggesting that they were derived from the common ancestor of arthropods.

### WSSV-related sequences and their implication in virus-host coevolution

So far no integration of virus, except the infectious hypodermal and hematopoietic necrosis virus (IHHNV), has been reported in the shrimp genome [20]. Our study is the first to demonstrate the prevalence of WSSV-like sequences in the *P. monodon *genome. Some of the WSSV-related PREs even reach a copy number in excess of 80,000 (= 400/0.45%) elements per genome. WSSV-related sequences have also been found in the genome of another penaeid species, *M. japonicus*[23]. Interestingly, although a number of shrimp viruses are prevalent in the wild, *e.g*., monodon baculovirus, hepatopancreatic parvovirus (HPV), and Taura Syndrome Virus (TSV) [50,51], WSSV seems to be the only virus of which integrated sequences heavily occupied the shrimp genome. Additionally, the WSSV-related sequences accumulated within the *P. monodon *genome appear to be restricted to only a number of WSSV genes, *e.g*., wsv514 (putative DNA polymerase III catalytic subunit), wsv447, wsv360 (structure protein, capsid), wsv332 (structure protein), wsv306 (structure protein, tegument), wsv289 (putative serine/threonine protein kinase), wsv209 (structure protein, envelop), and wsv037 (structure protein, capsid). Moreover, segments similar to some of these WSSV genes can be found in 2 or even 3 PREs. These WSSV-like sequences are thought to be continuously accumulated within the shrimp genomes perhaps by reinfection and/or by intracellular transposition.

One highly repeated WSSV-related PRE, FAM31&207, containing segments similar to wsv343 as well as IAP and Innexin 3, is of particular interest. IAPs, with the hallmark of 1-3 copies of a zinc-binding baculoviral IAP repeat (BIR) domains in its 5'-portion, are a conserved group of proteins that regulates apoptosis in both vertebrates and invertebrates [52]. In addition to survival, IAPs are thought to be important regulators in differentiation, innate immune response and cell motility [52]. The IAP-like sequence within the FAM31&207 shared 61% identity with the 5'-portion of the *P. monodon *IAP gene containing three BIR domains. Innexins, originally characterized as the structural proteins of gap junctions in fly and worm, are also members of an evolutionarily conserved large gene family [53]. The Innexin-like sequence within the FAM31&207 revealed 40% identity with the Innexin 3 gene of pea aphid (*Acyrthosiphon pisum*). IAP and WSSV-like sequences were also found in high redundancy in the genome of kuruma shrimp, *M. japonicus*[23]. Therefore, the hyper-expansion of IAP- and WSSV-like sequences, which might have arisen from segmental duplication events, is likely a common feature of penaeid genome.

Despite of their large quantity, the function of these WSSV-like sequences in the *P. monodon *genome is unclear. WSSV, as the sole species of a new virus family *Nimaviridae*, is a large dsDNA virus (~300 kb) with many unique characteristics on their genome and on morphology [54]. It displays a remarkably broad host range among crustaceans, but is highly pathogenic and virulent only on penaeid shrimps [54]. Complete WSSV genome analyses revealed that most of the WSSV-encoding proteins show no homology to known proteins, and the small number of genes with identifiable features (mainly involved in nucleotide metabolism and DNA replication) are more similar to eukaryotic than to viral genes [54]. Whether these WSSV-like sequences are remnants of the WSSVs integrating into the host genome, or instead belong to portions of the *P. monodon *genome subsequently acquired by the virus, remains unknown. These two possibilities may not be mutually exclusive. In the first scenario, the WSSV-like sequences present in the *P. monodon *genome resulted from WSSV integration. These WSSV-like sequences can exist as junk DNA of no particular consequence, or may affect the fitness of the host. Their multiplicity, which may facilitate nonhomologous recombination, implies that these WSSV-like segments play important roles in genome structure. In addition, some of them were shown transcriptionally active, indicating they might be functional. As mentioned above, a few WSSV genes accumulated many more copies than others in the *P. monodon *genome. One possible explanation is that selection for these specific WSSV genes to provide protection against infection of related exogenous pathogenic WSSV, *e.g*., by interfering their replication cycles, as demonstrated in the endogenous retroviruses (ERVs) in vertebrates [55] and in the endogenous rice tungro bacilliform virus (RTBV)-like sequences (ERTBVs) in rice [56].

In the second scenario, the WSSV-like sequences present in the *P. monodon *genome correspond to original parts of the host genome, which were subsequently gained by WSSV through horizontal transfers. For large DNA viruses that replicate in the nucleus of the host cell such as herpesvirus and baculovirus, the uptake of cellular genes into the viral genome may be of significant advantage [57]. In certain mammalian dsDNA viruses such as herpesvirus, the cellular homologues of virus assist in escaping from detection and destruction by the host immune system *via *imitating the structure and function of host genes [58]. This might be also the same for WSSV. A deeper investigation on the distribution and the fraction of the WSSV-like sequences in the genomes of other crustaceans with different susceptibility to WSSV is clearly needed, which will shed light on the role and evolution of WSSV-related sequences in the *P. monodon *genome.

## Conclusions

The high abundance of simple sequence repeats, novel transposable elements, and WSSV-like sequences illustrates the highly repetitive nature of the *P. monodon *genome. Especially, WSSV-like sequences, comprising over 20% of the *P. monodon *genome in length, highlights the uniqueness of genomic organization of penaeid shrimps from those of other arthropod lineages. Such a highly repetitive nature and the large genome size have placed major obstacles when working with the genomes of shrimp. The fosmid end sequences, along with the fosmid clone library, has provided the first glimpse into the sequence composition of an unsequenced crustacean genome, and will serve as a valuable resource for future physical mapping, whole genome sequencing and other genomic related studies.

## Methods

### Estimation of the genome size of *P. monodon*

The genome size of *P. monodon *was measured by flow cytometry of hemocytes. Samples were prepared according to the protocol of Chow *et al*. [7] with some modifications. Hemolymph was collected from the heart using a syringe containing 1 ml of phosphate-buffered saline-ribonuclease (PBS-RNase) solution (1% NaCl, 0.06% KCl, 0.0146% Na_2_HPO_4_, 0.004% KH_2_PO_4_, 1% sodium citrate, 2% sucrose, and 50 μg/ml RNase A). The hemolymph samples (approximately 1.1-1.5 ml) were transferred to Eppendorf tubes, held for 30 min at room temperature, and centrifuged at 600×*g *for 5 min. The pellet was resuspended in 1 ml of PBS-RNase solution, centrifuged, and resuspended in 0.3 ml of PBS. For fixation, 0.7 ml of ice-cold ethanol was gradually added to the cell suspension with gentle shaking. To remove cytoplasmic membrane, 0.05 ml of 1% (*v*/*v*) NP-40 solution was added, and the sample was vortexed for 3 times (2 sec per vortex). The sample was examined under a microscope to confirm the release of nuclei and then centrifuged at 600 ×*g *for 5 min. The pellet was resuspended in 1 ml of ice-cold 70% ethanol and filtered through 40-μm BD Falcon cell strainer (BD Biosciences) to remove debris and cell aggregates. The nuclei were stained by adding 0.01 ml of 0.1% (*w*/*v*) propidium iodide (PI) solution per 1 ml of sample. Then, by using flow cytometry (FC 500 System, Beckman Coulter) with an excitation wavelength of 488 nm and an emission wavelength of 615 nm, the fluorescence of 3,000-10,000 nuclei per sample was determined. The DNA distribution curves were analyzed by the WinMDI 2.8 software program (written by Joseph Trotter, Scripps Research Institute). DNA values were calculated by comparison to the human lymphocyte as a standard (3.50 pg DNA per nucleus) which were prepared by the same procedures but using the biological saline pH 7.4 as PBS instead.

### Fosmid library construction

A wild female tiger shrimp caught from the coastal waters of Taiwan was used as the DNA source. High-molecular weight DNA from the muscle was extracted by standard phenol-chloroform procedure. After this treatment most of the isolated DNA was blunt-ended and sheared in a size range of 40 to 50 kb. The DNA was end-repaired and ligated into the fosmid vector pCC1FOS according to the manufacturer's protocols (Copy Control™ Fosmid Production Kit; Epicentre Technologies). Fosmid clones were packaged using MaxPlax Lambda Packaging Extract. Packaged fosmid clones were stored at 4°C over chloroform in 1 ml of Phage Dilution Buffer (10 mM Tris-HCl at pH 8.3, 100 mM NaCl, 10 mM MgCl_2_). Well-separated colonies were picked, and were transferred into individual wells of 384 microtiter plates containing 60 μl/well LB supplemented with 10% glycerol and 12.5 μg/ml of chloramphenicol. The plates were incubated overnight at 37°C and then stored at -80°C.

### Size estimation of fosmid clones

To evaluate the average insert size in the library, 111 clones were randomly selected from the fosmid library. Fosmid clone DNA was isolated by a standard alkaline lysis method. The DNA was then completely digested using *Not*I (New England Biolabs) and subjected to pulsed-field gel electrophoresis (PFGE) (Rotaphor Typ V, Biometra) on 0.75% agarose gel in 0.3× Loening buffer (0.01 M Tris-HCl, 0.01 M NaH_2_PO_4_, 1 mM EDTA, pH 7.5). The gel-run parameters were as follows: initial voltage, 130 V; final voltage, 90 V; ramping, logarithmic; initial angle, 130°; final angle, 110°, ramping, linear; switch time: 2 sec.; run time, 14 h; temperature, 10C.

### Fosmid end sequencing

Fosmid DNA was isolated using Montage Plasmid MiniprepHTS kit (Millipore) according to the manufacturer's guidelines, and sequenced from both end with ABI BigDye Terminator v3.1 (Applied Biosystems) and ABI 3730xl DNA sequencer (Applied Biosystems). The forward sequencing primer sequence was 5'-GGATGTGCTGCAAGGCGATTAAGTTGG-3', and the reverse sequencing primer sequence was 5'-CTCGTATGTTGTGTGGAATTGTGAGC-3'. Base calling of chromatograms and trimming of fosmid-end sequences (FESs) were performed with PHRED software [59,60]. Vector sequence was masked with CROSS_MATCH http://www.genome.washington.edu and trimmed. Reads < 50 bp and phred score < 20 were eliminated from our internal end-sequence database.

### Repetitive sequence analysis

Fosmid end sequences were analyzed and masked with RepeatMasker [61] using default settings against *D. melanogaster *and *Anopheles gambiae *databases for the identification and annotation of repetitive sequences.

### Identification and characterization of microsatellites in the *P. monodon *genome

The program Tandem Repeat Finder [62] was performed, with the alignment parameters (match, mismatch, indels) {2, 3, 5} and score 50, to detect microsatellites (repeat unit 1-6 bp) of ≥ 12 bp within the 11,114,786-bp fosmid ends. To increase the stringency of this analysis, the tandem repeats identified with matches lower than or equal to 55% were manually removed from the final results.

### Novel repetitive elements

After known repetitive sequences were masked, the 20,926 fosmid end sequences were searched for tiger-shrimp-specific repetitive sequences by all *versus *all BlastN search, with parameters as described by Wiedmann *et al*. [63]. The BlastN results were fed to the RECON program [18] to *de novo *identify repetitive element families. The sequence families that repeated 20 or more times were identified. Repetitive sequence elements were further extracted from the sequence reads of each RECON family and reoriented according to RECON element definitions. A sequence alignment and a neighbor-joining tree were built, respectively, by MUSLE [64] and FastTree [65] for each repetitive element cluster. After thorough examination of all the alignments and the trees, one hundred and ninety-seven repetitive element families were obtained, with count numbers ranging from 10 to 971. The 197 repetitive sequence families were manually curated to either merge (due to sequence overlapping), or split (due to excessive sequence disparity). The families showing similarity to 18S or 28S ribosomal RNA genes and those that repeated less than 20 times were not analyzed further.

### Protein-coding sequences

To identify potential protein-coding regions, the 20,926 repeat-masked FESs were subjected to sequence similarity searches using BlastX on the NCBI *nr *protein database and BlastN on the Penaeus Genome Database http://sysbio.iis.sinica.edu.tw/page/[6], which contains over 200,000 Expressed Sequence Tags (ESTs) from four penaeid shrimps, *Penaeus monodon*, *Penaeus vannamei*, *Marsupenaeus japonicus*, and *Fenneropenaeus chinensis*. In BlastN analysis, a cutoff value of E-10 and a minimum match length of 100 bp with similarity threshold of ≥ 85% (for comparison with *P. monodon *ESTs) or ≥ 80% (for comparison with the ESTs of the other 3 penaeid species) were used. In BlastX analysis, a cutoff value of 1 × 10^-5 ^and a minimum match length of 300 bp were used as the similarity threshold.

## Authors' contributions

SH constructed the fosmid library, performed the fosmid-end-sequence analysis and drafted the manuscript. YL and EY assisted in the fosmid-end-sequence analysis. TL, HS, KW, and ST assisted in the fosmid end sequencing. CL and GK provided the EST data. GM and MC measured the DNA content with the flow cytometry to determine the genome size. DW performed the self-BlastN analysis to identify the novel repetitive element families. TA and IH assisted in the fosmid library construction. HY conceived of the study and participated in its design and coordination. All authors read and approved the final manuscript.

## Acknowledgements

We thank Wen-Hsiung Li, Manyuan Long, Kevin Bullaughey and two anonymous reviewers for their helpful comments. We also thank Chung-Yen Lin and his lab for technical support in bioinformatic analysis. Financial support was granted to Hon-Tsen Yu by the National Science Council of Taiwan, ROC (952317B002008 and 962317B002012), and the National Taiwan University, ROC (97R006628 and 98R006628).

## Supplementary Material

Additional file 1 **Estimates of the *P. monodon *genome size, as percentage of human DNA**. Click here for file

Additional file 2 **Insert sizes of representative clones from the *P. monodon *fosmid library**. The first and last lanes of each gel are 36 kb-size markers. The average insert size is 40.8 kb. Click here for file

Additional file 3 **Frequency and length distribution of the 20,926 *Penaeus monodon *fosmid end sequences**. Click here for file

Additional file 4 **Examples of *P. monodon *ESTs found to contain a very long stretch of microsatellites (a). Some sets of ESTs derived from the same gene showed copy number variation in the microsatellites they contain (b)**. Dinucleotide repeats [(TC)_50_, (TA) _50_, (TG)_50_, and (CG)_50_] were used as query sequences to search against the Penaeus Genome Database. Only top 10 hits were listed. Click here for file

Additional file 5 **Thirty-six PREs were found transcriptionally active *via *BlastN search against the *P. monodon *EST dataset (PmTwN) in the Penaeus Genome Database**. Only top 3 hits are listed. Click here for file
