# Supplementary material for: Fosmid library end sequencing reveals a rarely known genome structure of marine shrimp Penaeus monodon
Source: BMC Genomics. 2011 May 17;12:242. doi: 10.1186/1471-2164-12-242 (PMC3124438; doi:10.1186/1471-2164-12-242)
Supplement: Additional file 1 — Estimates of the P. monodon genome size, as percentage of human DNA. [file 1471-2164-12-242-S1.DOC]

**Additional file 1.** **Estimates of the *P. monodon* genome size, as percentage of human DNA.**

|  |  |  | Peak of 2*n* | |
| --- | --- | --- | --- | --- |
| Species | Sample | Number of cells analyzed | DNA content ±SD (%) | CV (%)^*^ |
| Human | 2 ♂ | 31,958 | 100 | — |
| *P. monodon* | 6 ♂, 15♀ | 144,177 | 72.2±5.0 | 6.93 |
| *P. vannamei* | 2 ♂, 2♀ | 33,448 | 71.5±0.4 | 0.56 |

^*^CV, coefficient of variation
