# Supplementary material for: Fosmid library end sequencing reveals a rarely known genome structure of marine shrimp Penaeus monodon
Source: BMC Genomics. 2011 May 17;12:242. doi: 10.1186/1471-2164-12-242 (PMC3124438; doi:10.1186/1471-2164-12-242)
Supplement: Additional file 2 — Insert sizes of representative clones from the P. monodon fosmid library. The first and last lanes of each gel are 36 kb-size markers. The average insert size is 40.8 kb. [file 1471-2164-12-242-S2.PPT]

## Slide 1
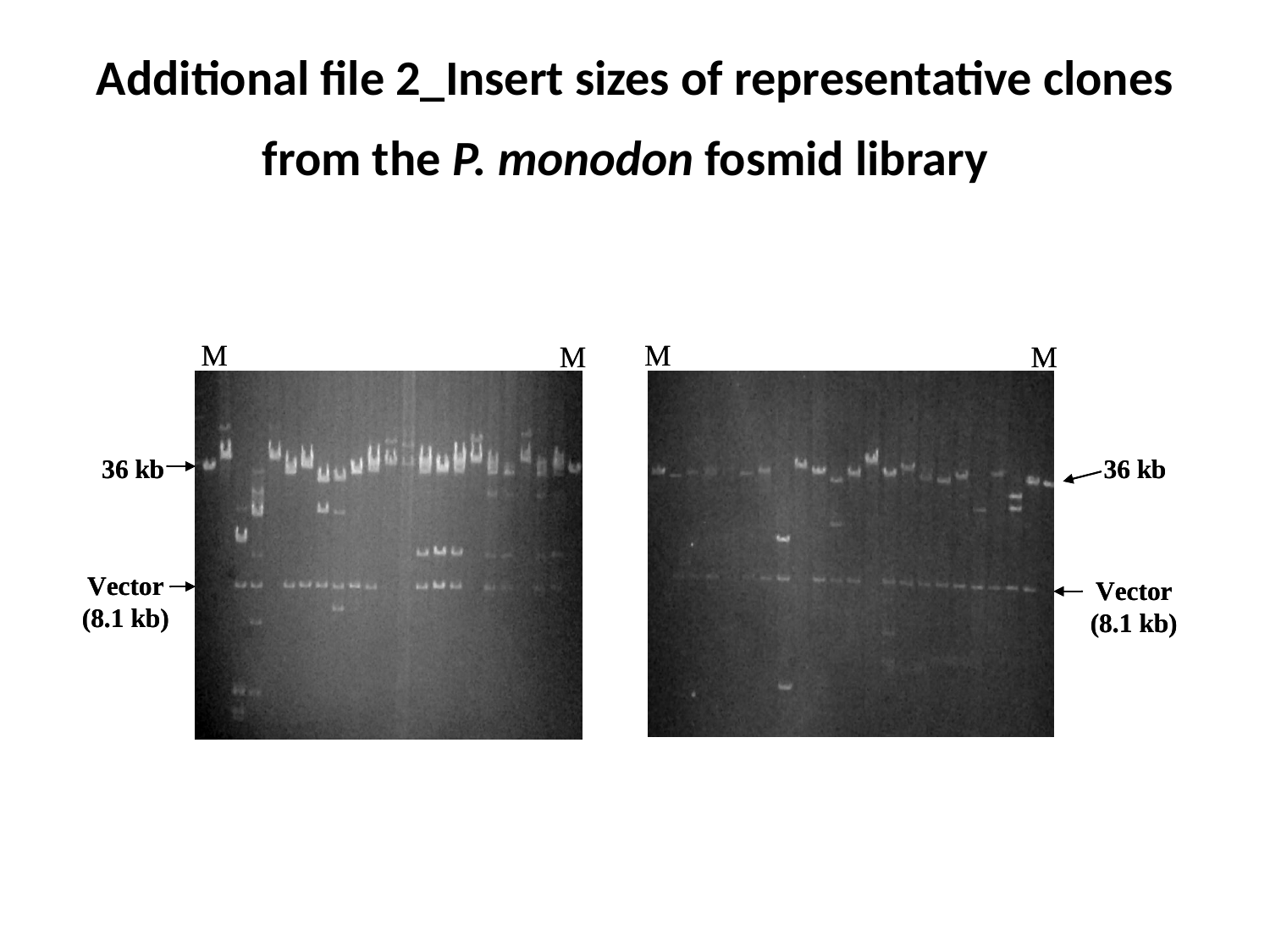

# Additional file 2_Insert sizes of representative clones from the P. monodon fosmid library
