# Supplementary material for: Fosmid library end sequencing reveals a rarely known genome structure of marine shrimp Penaeus monodon
Source: BMC Genomics. 2011 May 17;12:242. doi: 10.1186/1471-2164-12-242 (PMC3124438; doi:10.1186/1471-2164-12-242)
Supplement: Additional file 3 — Frequency and length distribution of the 20,926 Penaeus monodon fosmid end sequences. [file 1471-2164-12-242-S3.DOC]

**Additional file 3. Frequency and length distribution of *Penaeus monodon*** fosmid end sequences

| **Range (bp)** | **Frequency (%)** | **Average (bp)** | **GC%** | **Total length (bp)** |
| --- | --- | --- | --- | --- |
| 100-199 | 1,877 (9%) | 145 | 44.5 | 272,673 |
| 200-299 | 1,466 (7%) | 249 | 44.3 | 364,449 |
| 300-399 | 1,390 (7%) | 348 | 44.9 | 484,378 |
| 400-499 | 1,627 (8%) | 452 | 44.6 | 736,105 |
| 500-599 | 3,194 (15%) | 559 | 45.9 | 1,785,243 |
| 600-699 | 10,407 (50%) | 651 | 46.4 | 6,779,783 |
| 700-899 | 965 (4%) | 717 | 42.6 | 692,155 |
| **Total** | **20,926 (100%)** | **531** | **45.6** | **11,114,786** |
