# Supplementary material for: Fosmid library end sequencing reveals a rarely known genome structure of marine shrimp Penaeus monodon
Source: BMC Genomics. 2011 May 17;12:242. doi: 10.1186/1471-2164-12-242 (PMC3124438; doi:10.1186/1471-2164-12-242)
Supplement: Additional file 4 — Examples of P. monodon ESTs found to contain a very long stretch of microsatellites (a). Some sets of ESTs derived from the same gene showed copy number variation in the microsatellites they contain (b). Dinucleotide repeats [(TC)50, (TA) 50, (TG)50, and (CG)50] were used as query sequences to search against the Penaeus Genome Database. Only top 10 hits were listed. [file 1471-2164-12-242-S4.DOC]

**Additional file 4**. **Examples of *P. monodon* ESTs found to contain a very long stretch of microsatellites (a). Some sets of ESTs derived from the same gene showed copy number variation in the microsatellites they contain (b).** Dinucleotide repeats [(TC)_50_, (TA) _50_, (TG)_50_, and (CG)_50_] were used as query sequences to search against the Penaeus Genome Database. Only top 10 hits were listed.

(a)

| **EST Contig** | **Contig length (bp)** | **Dominant repeat motif** | **Sequence** |
| --- | --- | --- | --- |
| 6687_TUS_5283 | 479 | TC | (TCTCTT)_3_(TCTTTC)_2_(TTTCTC)_3_(TTTCTT)_2_**(TC)_87_** |
| 6687_TUS_4566 | 282 | TC | **(TC)_3_**(GC)**(TC)_56_** |
| 6687_TUS_4921 | 328 | AG | **(AG)_3_**(GA)**(AG)_49_**(CG)**(AG)_9_**(AA)**(AG)_8_** |
| 6687_TUS_4629 | 487 | AG | **(AG)_3_**(TG)**(AG)_7_**…6 bp...**(AG)_39_**(AA)**(AG)_11_**(G)**(AG)_36_**(AA)**(AG)_9_**(G)**(AG)_28_**(A)**(AG)_7_** |
| 6687_TUS_4998 | 342 | GA | **(GA)_11_**(AAGG)**(GA)_11_**…6 bp…**(GA)_36_**(A)**(GA)_14_** |
| 6687_TUS_5403 | 760 | TC | **(TC)_2_**(AC)**(TC)_48_** |
| 6687_TUS_5277 | 486 | CT | [**(CT)_2_**CA]**_2_(CT)_3_**(CA)**(CT)_3_**(CA)**(CT)_3_**(CA)(CTCCCT)**(CT)_27_**(CC)**(CT)_13_**(CC)**(CT)_15_**(GT)**(CT)_2_** |
| 6687_TUS_5147 | 320 | GA | **(GA)_3_**(AA)**(GA)_3_**(GT)**(GA)_46_** |
| 6687_TUS_4751 | 315 | TC | **(TC)_33_**(TG)**(TC)_3_**(TG)**(TC)_21_** |
| 6687_TUS_3878 | 304 | AG | **(AG)_15_**(GG)**(AG)_35_** |
| 6687_TUS_5263 | 422 | AT | **(AT)_47_**(GT)_6_(ATGC)_2_(ACGC)(GT)_12_…19 bp ... (GC)_8_..41 bp....(GA)_7_ |
| 6687_TUS_4528 | 159 | AT | **(AT)_6_**(GT)**(AT)_42_**(AC)**(AT)_3_** |
| 6687_TUS_7398 | 622 | TA | **(TA)_42_**(T)_4_ **(TA)_8_**…26 bp .. **(TA)_7_**…34 bp…**(TA)_12_**…7 bp… **(AT)_5_** |
| 6687_TUS_5393 | 719 | TA | **(TA)_47_**…6 bp .. **(TA)_19_** |
| 6687_TUS_5717 | 513 | TA | **(TA)_33_**(TG)**(TA)_9_**(TC)**(TA)_12_**(CA)**(TA)_5_**(GCA)**(TA)_23_** |
| 6687_TUS_5145 | 401 | TA, TG | **(TA)_48_**(TG)**(TA)_3_**T**(TG)_15_** |
| 6687_TUS_4667 | 327 | AT | **(AT)_9_**(GT)**(AT)_20_**...74 bp....(**AT)_22_**.. ..22 bp.... **(AT)_51_** |
| 6687_TUS_4893 | 705 | TA | **(TA)_55_** |
| 6687_TUS_6336 | 425 | AT | **(AT)_50_**(CT)**(AT)_4_** |
| 6687_TUS_6667 | 463 | TA | **(TA)_66_**...41 bp.... **(TA)_14_**...9 bp.... **(TA)_13_** |
| 6687_TUS_6563 | 280 | AC | **(AC)_8_**(AT)**(AC)_7_**(CAA)**(AC)_7_**…4 bp ...**(AC)_28_** |
| 6687_TUS_4607 | 345 | AC | [**(AC)_2_**GC]**(AC)_9_**[**(AC)_2_**GC]**_10_ (AC)_27_**[**(AC)_2_**GC]**_2_(AC)_3_**(GC)**(AC)_11_** |
| 6687_TUS_5395 | 695 | AC, AT | **(AC)_12_**(AAAT)**(AC)_4_**(AT)**(AC)_7_**(AT)**(AC)_26_**(TC)**(AT)_30_**…13 bp...**(AT)_6_**…38 bp...**(AT)_6_**…8 bp... **(AT)_8_**…17 bp...**(AT)_4_**(GT)**(AT)_4_** |
| 6687_TUS_6875 | 448 | GT | **(GT)_25_**…8 bp ...**(GT)_12_**(GA)**(GT)_5_**(GC)**(GT)_6_** |
| 6687_TUS_4650 | 494 | GA, CA | **(GA)_4_**…16 bp ...**(GA)_19_**...207 bp...**(CA)_44_** |
| 6687_TUS_7226 | 311 | TG | **(TG)_47_** |
| 6687_TUS_3117 | 561 | AC | **(AC)_46_**(AA)**(AC)_2_** |
| 6687_TUS_4828 | 211 | AC, TA | **(AC)_7_**...**(AC)_47_**....55 bp...**(TA)_21_** |
| 6687_TUS_4774 | 262 | AC | **(AC)_2_**(AG)**(AC)_43_**(GC)**(AC)_19_** |
| 6687_TUS_5398 | 198 | CA | **(CA)_78_** |
| 6687_TUS_4835 | 169 | TA, GC | **(TA)_27_**(TG)**(TA)_5_**...51 bp...**(GC)_14_** |
| 6687_TUS_4601 | 542 | TA, CA, GC | **(TA)_7_**(AA)**(TA)_33_(CA)_17_**....51 bp....**(GC)_19_** |
| 6687_TUS_5389 | 411 | CA, TA, TG, GC | **(CA)_16_(TA)_14_(TG)_20_**...21 bp...**(GC)_18_**(GTGC)**(GT)_8_**...7 bp...**(AT)_11_(GT)_6_**. ..12 bp...(GA)_8_ |
| 6687_TUS_4674 | 589 | TA, CG | **(TA)_17_**…44 bp...**(CG)_13_**…**(CG)_4_** |
| 6687_TUS_4797 | 293 | TA, GC | **(TA)_3_**…8 bp... **(TA)_19_**(TG)_8_(AG)_8_**(AT)_20_**(A)**(GC)_20_**(GT)_2_(GC)(GT)_7_(GA)_4_(CA)_6_ |
| 6687_TUS_4855 | 182 | AT, GC | **(AT)_30_**(A)_9_(GA)_7_**(GC)_20_**(GTGC)(GT)_6_(GA)_6_ |
| 6687_TUS_5153 | 403 | TA, GC, GT | **(TA)_21_**(GA)**(TA)_4_**(GA)**(TA)_2_**(GA)**(TA)_3_**(GA)**(TA)_4_**(GA)**(TA)_3_**...5 bp...**(GC)_21_(GT)_18_** |

^*^ Dinucleotide repeats [(TC)_50_, (TA) _50_, (TG)_50_, and (CG)_50_] were used as query sequences to search against the Penaeus Genome Database. Only top 10 hits were listed.

(b)

| **EST contig** | **ESTs included** | **Sequence** | **Annotation [species]** | **E-value; Identity** |
| --- | --- | --- | --- | --- |
| 6687_TUC_1023 | | (AT)_5-8_...(AT)_2-4_...(AT)_11-26_…..(AT)_2-10_ | – | – |
|  | GO079142 | (AT)_8_….(AT)_4_…..(AT)_20_…….(AT)_10_ |  |  |
|  | GO078700 | (AT)_8_ |  |  |
|  | GO075996 | (AT)_7_….(AT)_2_…..(AT)_24_.........(AT)_7_ |  |  |
|  | GO072770 | (AT)_8_……………(AT)_11_ |  |  |
|  | GO076957 | (AT)_5_……………(AT)_26_ |  |  |
|  | GO075762 | (AT)_8_…………....(AT)_4_ |  |  |
| 6687_TUC_1538 | | (TAA)_20-22_ | *C-type lectin 3* [*F. chinensis*] | 9e-25; 53/132 (41%) |
|  | DT366714 | (TAA)_22_ |  |  |
|  | GO075269 | (TAA)_21_ |  |  |
|  | GO077396 | (TAA)_22_ |  |  |
|  | GO076822 | (TAA)_20_ |  |  |
|  | EE662819 | (TAA)_22_ |  |  |
| 6687_TUC_516 | | (TA)_22-27_….84 bp…..(AT)_13-26_ | – | – |
|  | GO069377 | (TA)_27_….....84 bp…..(AT)_26_ |  |  |
|  | GO073360 | (TA)_26_….....84 bp…..(AT)_25_ |  |  |
|  | GO078343 | (TA)_22_….....84 bp..…(AT)_13_ |  |  |
|  | GO070164 | (TA)_26_….....84 bp…..(AT)_26_ |  |  |
|  | GO067273 | (TA)_27_….....84 bp..…(AT)_25_ |  |  |
|  | GO070064 | (TA)_27_….....84 bp..…(AT)_26_ |  |  |
|  | GO068832 | (TA)_25_…….84 bp..…(AT)_26_ |  |  |
