# Supplementary material for: Fosmid library end sequencing reveals a rarely known genome structure of marine shrimp Penaeus monodon
Source: BMC Genomics. 2011 May 17;12:242. doi: 10.1186/1471-2164-12-242 (PMC3124438; doi:10.1186/1471-2164-12-242)
Supplement: Additional file 5 — Thirty-six PREs were found transcriptionally active via BlastN search against the P. monodon EST dataset (PmTwN) in the Penaeus Genome Database. Only top 3 hits are listed. [file 1471-2164-12-242-S5.DOC]

**Additional file 5**. **Thirty-six PREs were found transcriptionally active via BlastN search against the *P. monodon* EST dataset (PmTwN) in the Penaeus Genome Database.** Only top 3 hits are listed.

(i) WSSV-related (14 PREs)

| **PRE** | **ESTs** | **Probability** | **Identity** |
| --- | --- | --- | --- |
| FAM9_15-44 | EE724281 | 0 | 586/ 600 (98%) |
|  | ES-N-S03-0558-W | 0 | 574/587 (98%) |
|  | DW405577 | 0 | 412/414 (100%) |
| FAM152 | HPO-N-S01-0227-LF | 0 | 582/600 (97%) |
|  | DW404942 | 0 | 491/492 (100%) |
|  | ES-N-S02-0311-W | 0 | 491/494 (99%) |
| FAM2 | DW405238 | 0 | 747/751 (99%) |
|  | ES-N-S03-0033-W | 0 | 598/600 (100%) |
|  | LP-N-S01-0389-LF | 0 | 513/513 (100%) |
| FAM1 | EE724341 | 0 | 600/628 (96%) |
|  | ES-N-S03-0135-W | 0 | 436/457 (95%) |
|  | DW405302 | 0 | 429/449 (96%) |
| FAM31&207 | HPO-N-S01-0705-LF | 0 | 599/600 (100%) |
|  | ES-N-S03-0380-W | 0 | 552/563 (98%) |
|  | DW405427 | 0 | 547/556 (98%) |
| FAM87 | DW405003 | 0 | 695/697 (100%) |
|  | ES-N-S02-0419-W | 0 | 598/600 (100%) |
|  | DW404951 | 0 | 394/397 (99%) |
| FAM24 | DW405074 | 0 | 637/674 (95%) |
|  | ES-N-S02-0187-W | 0 | 572/600 (95%) |
| FAM5 | EE724282 | 0 | 542/555 (98%) |
| FAM43 | EB389114 | 0 | 726/734 (99%) |
|  | LP-Y-S01-0073-LF | 0 | 597/601 (99%) |
| FAM361 | ES-N-S03-0003-W | 0 | 482/526 (92%) |
|  | DW405217 | 0 | 420/446 (94%) |
| FAM259 | DW404879 | 0 | 641/642 (100%) |
|  | ES-N-S02-0077-W | 0 | 600/600 (100%) |
|  | EE724276 | 0 | 602/611 (99%) |
| FAM137 | DW404900 | 0 | 707/715 (99%) |
|  | ES-N-S02-0039-W | 0 | 591/600 (99%) |
| FAM158 | DW405141 | 0 | 602/608 (99%) |
|  | ES-N-S02-0276-W | 0 | 595/602 (99%) |
| FAM139 | HPO-N-S01-0991-LF | 1.10E-112 | 228/232 (98%) |
|  | DY542629 | 1.00E-110 | 217/218 (99%) |

(ii) Retrotransposon-related (10 PREs)

| **PRE** | **ESTs** | **Probability** | **Identity** |
| --- | --- | --- | --- |
| FAM9_1-14 | [DW404968](http://sysbio.iis.sinica.edu.tw/page/shrimp_db/est_info.php?est_id=18448) | 0 | 723/766 (94%) |
|  | [EE662012](http://sysbio.iis.sinica.edu.tw/page/shrimp_db/est_info.php?est_id=16811) | 0 | 719/765 (94%) |
|  | [DW405245](http://sysbio.iis.sinica.edu.tw/page/shrimp_db/est_info.php?est_id=18171) | 0 | 709/774 (92%) |
| FAM185 | [DW405159](http://sysbio.iis.sinica.edu.tw/page/shrimp_db/est_info.php?est_id=18257) | 0 | 756/786 (96%) |
|  | [DY542501](http://sysbio.iis.sinica.edu.tw/page/shrimp_db/est_info.php?est_id=17399) | 0 | 748/799 (94%) |
|  | [EE661814](http://sysbio.iis.sinica.edu.tw/page/shrimp_db/est_info.php?est_id=17009) | 0 | 711/767 (93%) |
| FAM309 | [HPO-N-S01-0774-LF](http://sysbio.iis.sinica.edu.tw/page/shrimp_db/est_info.php?est_id=) | 3.60E-107 | 214/216 (99%) |
| FAM189_7~16 | [ES-N-S02-0444-W](http://sysbio.iis.sinica.edu.tw/page/shrimp_db/est_info.php?est_id=) | 2.30E-113 | 226/228 (99%) |
|  | [DW405017](http://sysbio.iis.sinica.edu.tw/page/shrimp_db/est_info.php?est_id=18399) | 8.10E-113 | 225/227 (99%) |
| FAM75_17-25,35-36,39-40 | [HPO-N-S01-0619-LF](http://sysbio.iis.sinica.edu.tw/page/shrimp_db/est_info.php?est_id=) | 0 | 539/547 (99%) |
|  | [EE724285](http://sysbio.iis.sinica.edu.tw/page/shrimp_db/est_info.php?est_id=15030) | 0 | 526/568 (93%) |
|  | [EE724334](http://sysbio.iis.sinica.edu.tw/page/shrimp_db/est_info.php?est_id=14981) | 0 | 458/501 (91%) |
| FAM18 | [EE724253](http://sysbio.iis.sinica.edu.tw/page/shrimp_db/est_info.php?est_id=15061) | 2.20E-97 | 285/336 (85%) |
| FAM75_1-9, 11-16 | [DW404885](http://sysbio.iis.sinica.edu.tw/page/shrimp_db/est_info.php?est_id=18531) | 0 | 704/756 (93%) |
|  | [PmTwI01A05.scf_3](http://sysbio.iis.sinica.edu.tw/page/shrimp_db/est_info.php?est_id=) | 0 | 665/698 (95%) |
|  | [HC-W-S01-0330-LF](http://sysbio.iis.sinica.edu.tw/page/shrimp_db/est_info.php?est_id=) | 0 | 569/601 (95%) |
| FAM9_45-54 | [DY542549](http://sysbio.iis.sinica.edu.tw/page/shrimp_db/est_info.php?est_id=17351) | 0 | 601/633 (95%) |
|  | [HPO-N-S01-0581-LF](http://sysbio.iis.sinica.edu.tw/page/shrimp_db/est_info.php?est_id=) | 0 | 571/602 (95%) |
|  | DW405048 | 0 | 651/711 (92%) |
| FAM393 | [DW405409](http://sysbio.iis.sinica.edu.tw/page/shrimp_db/est_info.php?est_id=18007) | 0 | 441/465 (95%) |
|  | [ES-N-S03-0354-W](http://sysbio.iis.sinica.edu.tw/page/shrimp_db/est_info.php?est_id=) | 0 | 461/498 (93%) |
|  | [HC-V-S01-0380-LF](http://sysbio.iis.sinica.edu.tw/page/shrimp_db/est_info.php?est_id=) | 0 | 485/556 (87%) |
| FAM1106 | [FK826366](http://sysbio.iis.sinica.edu.tw/page/shrimp_db/est_info.php?est_id=14264) | 0 | 521/549 (95%) |

(iii) Annotated (3 PREs)

| **PRE** | **ESTs** | **Probability** | **Identity** |
| --- | --- | --- | --- |
| FAM327 | [EE724298](http://sysbio.iis.sinica.edu.tw/page/shrimp_db/est_info.php?est_id=15017) | 0 | 390/392 (99%) |
| FAM575 | [ES-N-S02-0151-W](http://sysbio.iis.sinica.edu.tw/page/shrimp_db/est_info.php?est_id=) | 2.40E-72 | 186/205 (91%) |
| FAM46 | [EE724316](http://sysbio.iis.sinica.edu.tw/page/shrimp_db/est_info.php?est_id=14999) | 1.90E-175 | 358/368 (97%) |

(iv) Unannotated (9 PREs)

| **PRE** | **ESTs** | **Probability** | **Identity** |
| --- | --- | --- | --- |
| FAM72 | [PmTwI15G05.scf_3](http://sysbio.iis.sinica.edu.tw/page/shrimp_db/est_info.php?est_id=) | 1.00E-95 | 260/293 (89%) |
|  | [PmTwI15G05.ab1_5](http://sysbio.iis.sinica.edu.tw/page/shrimp_db/est_info.php?est_id=) | 2.20E-92 | 218/237 (92%) |
| FAM67 & 606 & 707 | [HC-N-S01-0160-LF](http://sysbio.iis.sinica.edu.tw/page/shrimp_db/est_info.php?est_id=) | 0 | 625/731 (85%) |
|  | [DW042583](http://sysbio.iis.sinica.edu.tw/page/shrimp_db/est_info.php?est_id=20878) | 0 | 625/731 (85%) |
|  | [HPO-N-S01-0828-LF](http://sysbio.iis.sinica.edu.tw/page/shrimp_db/est_info.php?est_id=) | 9.50E-150 | 300/310 (97%) |
| FAM188 | GE616455 | 7.30E-141 | 338/369 (92%) |
|  | [ES-N-S03-0444-W](http://sysbio.iis.sinica.edu.tw/page/shrimp_db/est_info.php?est_id=) | 7.40E-136 | 344/380 (91%) |
|  | [DW405467](http://sysbio.iis.sinica.edu.tw/page/shrimp_db/est_info.php?est_id=17949) | 7.40E-136 | 344/380 (91%) |
| FAM57 | [DW405124](http://sysbio.iis.sinica.edu.tw/page/shrimp_db/est_info.php?est_id=18292) | 0 | 520/546 (95%) |
|  | ES-N-S02-0257-W | 1.00E-177 | 356/373 (95%) |
|  | HPO-N-S01-0399-LF | 4.00E-69 | 157/169 (92%) |
| FAM632 | [HPO-N-S01-0811-LF](http://sysbio.iis.sinica.edu.tw/page/shrimp_db/est_info.php?est_id=) | 0 | 558/573 (97%) |
| FAM839 | [EE724335](http://sysbio.iis.sinica.edu.tw/page/shrimp_db/est_info.php?est_id=14980) | 0 | 513/521 (98%) |
|  | EE724265 | 0 | 498/506 (98%) |
| FAM244 | [DW405131](http://sysbio.iis.sinica.edu.tw/page/shrimp_db/est_info.php?est_id=18285) | 0 | 633/642 (99%) |
|  | ES-N-S02-0265-W | 0 | 634/655 (96%) |
| FAM569 | [DW405389](http://sysbio.iis.sinica.edu.tw/page/shrimp_db/est_info.php?est_id=18027) | 0 | 405/405 (100%) |
|  | ES-N-S03-0308-W | 0 | 405/406 (99%) |
|  | HPO-N-S01-0836-LF | 1.00E-117 | 231/232 (99%) |
| FAM696 | [HPO-N-S01-0374-LF](http://sysbio.iis.sinica.edu.tw/page/shrimp_db/est_info.php?est_id=) | 0 | 547/592 (92%) |

***^a^*** Cut-off value : 1E-40; matched length: 200 bp; identity: 85%.
